# Inhibitory influence of three new synthesized cationic gemini surfactants on the corrosion rate of carbon steel in 1 M HCl

**DOI:** 10.1038/s41598-026-44281-2

**Published:** 2026-04-09

**Authors:** Fatma M. Abdelhafiz, Radwa M. Sami, Eman A. Ghiaty, Ashgan I. Awad

**Affiliations:** Petrochemicals Department, Egyptian Petroleum Research Institute (EPRI), Nasr City, Cairo Egypt

**Keywords:** Cationic gemini inhibitor surfactants, Micellization, Surface properties, Schiff base derivatives, Chemistry, Materials science

## Abstract

Carbon steel is a fundamental material used across numerous industries; however, it is highly susceptible to corrosion when exposed to various media, particularly mineral acids that are widely employed in industrial processes. Surfactants capable of adsorbing into metal surfaces act as effective organic corrosion inhibitors. Owing to their inherent surface activity in aqueous environments, these compounds can provide efficient and simultaneous protection of metal substrates. In this study, three novels cationic gemini inhibitor surfactants (CGIS) were synthesized through a multi-step procedure involving Schiff base formation followed by quaternization reactions. The synthesized compounds IIIa, IIIb and IIIc were obtained in high purity with good yields. Structural characterization of the surfactants was carried out using Fourier Transform Infrared (FTIR) spectroscopy and Proton Nuclear Magnetic Resonance (^1^H NMR) spectroscopy. The corrosion inhibition performance of CGIS for carbon steel was evaluated in 1.0 M HCl acidic medium was systematically investigated. The evaluation employed a combination of gravimetric (weight loss) measurements, electrochemical impedance spectroscopy (EIS), and potentiodynamic polarization techniques. The results of this study indicate that increasing the CGIS concentration and temperature leads to enhanced inhibition efficiency. The improved performance is attributed to the adsorption of CGIS molecules into the carbon steel (CS) surface, forming a protective barrier against corrosion. Potentiodynamic polarization measurements revealed that CGIS acts as a mixed-type inhibitor. Changes observed in electrochemical impedance spectra confirmed the formation of a protective layer resulting from inhibitor adsorption on the CS surface. The adsorption behavior of CGIS on carbon steel was found to follow the Langmuir adsorption isotherm. Furthermore, kinetic and thermodynamic parameters were calculated and analyzed to elucidate the inhibition mechanism. Scanning electron microscopy (SEM) images further demonstrated the improved surface morphology of carbon steel in the presence of CGIS.

**Supplementary Information:**

The online version contains supplementary material available at 10.1038/s41598-026-44281-2.

## Introduction

Carbon steel, a commonly used metal material, is valued for its high plasticity, superb welding performance, and superior mechanical qualities, making it useful in a variety of industries^1^. Carbon steel has numerous applications, including pipelines, petroleum, metal processing equipment, and electrochemical sectors^2^. Carbon steel can be cleaned in an acid bath, a procedure known as pickling, to remove rust and oxides from the metal surface. Mineral acids such as sulfuric acid, hydrochloric acid, and so on are commonly employed. Metals and alloys degrade due to corrosion, necessitating the replacement of various machine components.

Corrosion of carbon steel pipelines and infrastructure is a significant challenge in oil and gas transportation, especially in environments with acidic conditions, high salinity, or the presence of CO_2_ and H_2_S. This deterioration is primarily an electrochemical process, where the metal serves as an anode and undergoes oxidation, while a cathodic reaction—often involving the reduction of oxygen or hydrogen ions—occurs simultaneously^3^. Such corrosion greatly compromises the structural integrity and operational lifespan of pipeline systems. In response, organic corrosion inhibitors have garnered attention as effective alternatives. These inhibitors can adsorb onto metal surfaces, forming a protective barrier that hinders metal dissolution^4^.

Surfactant molecules are particularly promising as corrosion inhibitors, as they help create a protective layer on the metal surface^5,6^. Organic surfactants reduce the rates of anodic and cathodic reactions by blocking active sites through adsorption on the metal surface^7^. The amphiphilic characteristics of surfactants are believed to enhance their adsorption at the solid/liquid interface^8^, effectively blocking reactive sites that are susceptible to degradation. Additionally, substituted pyridinium-based compounds tend to be less toxic than their unsubstituted counterparts and have various biological applications^9^. Gemini surfactants, in particular, have demonstrated considerable potential as corrosion inhibitors^10^.

The main anti-corrosion mechanism of gemini surfactants is the formation of a compact, dense protective layer on the metal surface. This layer serves as a physical barrier, effectively preventing aggressive species, such as chloride or hydrogen ions, from reaching the metal and significantly reducing the corrosion rate. Adsorption can occur through physisorption, which involves weak van der Waals forces or electrostatic attraction, or through chemisorption, where electrons are shared or transferred between the gemini molecule and the metal surface. The specific adsorption mechanism depends on the surfactant structure and the corrosive environment^10–12^.

In this paper, cationic gemini surfactants were prepared, distinguished by their various hydrophobic tails using the Schiff base reaction. A comprehensive study was conducted on the influence of hydrophobic tail length on surfactant structural characteristics. Additionally, the impact of the hydrophobic tail and temperature on surface and thermodynamic properties was investigated. The influence of the hydrophobic tail’s length on the functionality of the synthesized compounds was studied, followed by an analysis of their surface parameters. Effective corrosion protection was provided by organic inhibitors, which were also evaluated for their environmental safety as anticorrosion agents.

## Materials and methods

### Materials

(Glyoxal, ethylene diamine, 3-acetylpyridine,1-octylbromide, 1-dodecyl bromide, 1-hexadecyl bromide) had been purchased from Sigma-Aldrich, Methanol and Ethanol and other materials were sourced from ADWIC Company.

### Preparation method

**Fig. 1:**
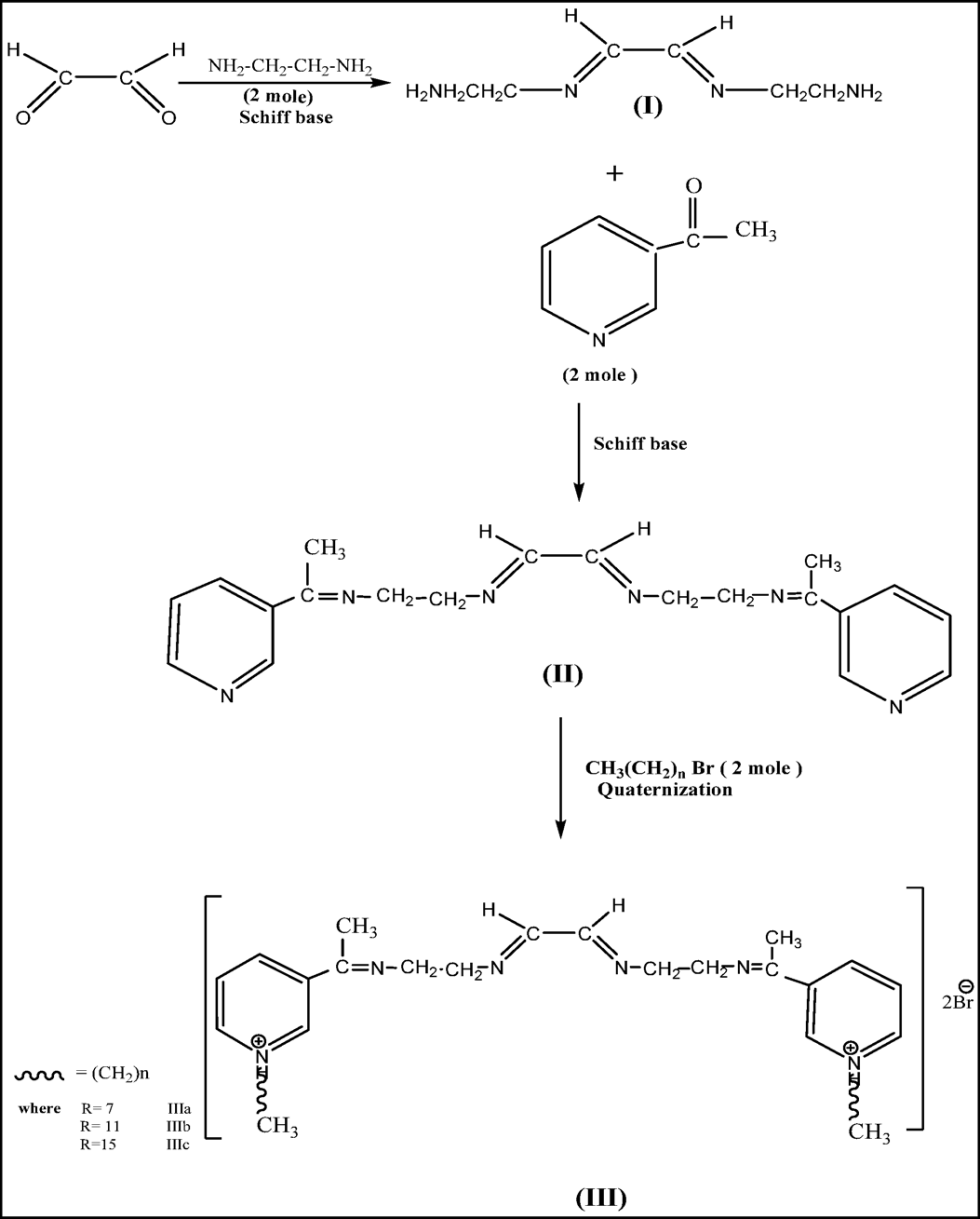
Synthesis of cationic gemini inhibitor surfactants (CGIS) IIIa, IIIb, and IIIc.

Preparation of three Cationic gemini inhibitor surfactants -based on Schiff base and quaternization reactions were prepared through three steps using two steps Schiff base reaction followed by quaternization reaction step. to obtain (IIIa, IIIb, IIIc) Illustrated As shown in Fig. 1.

#### Synthesizing cationic gemini inhibitor surfactants (CGIS)

**Fig. 2:**
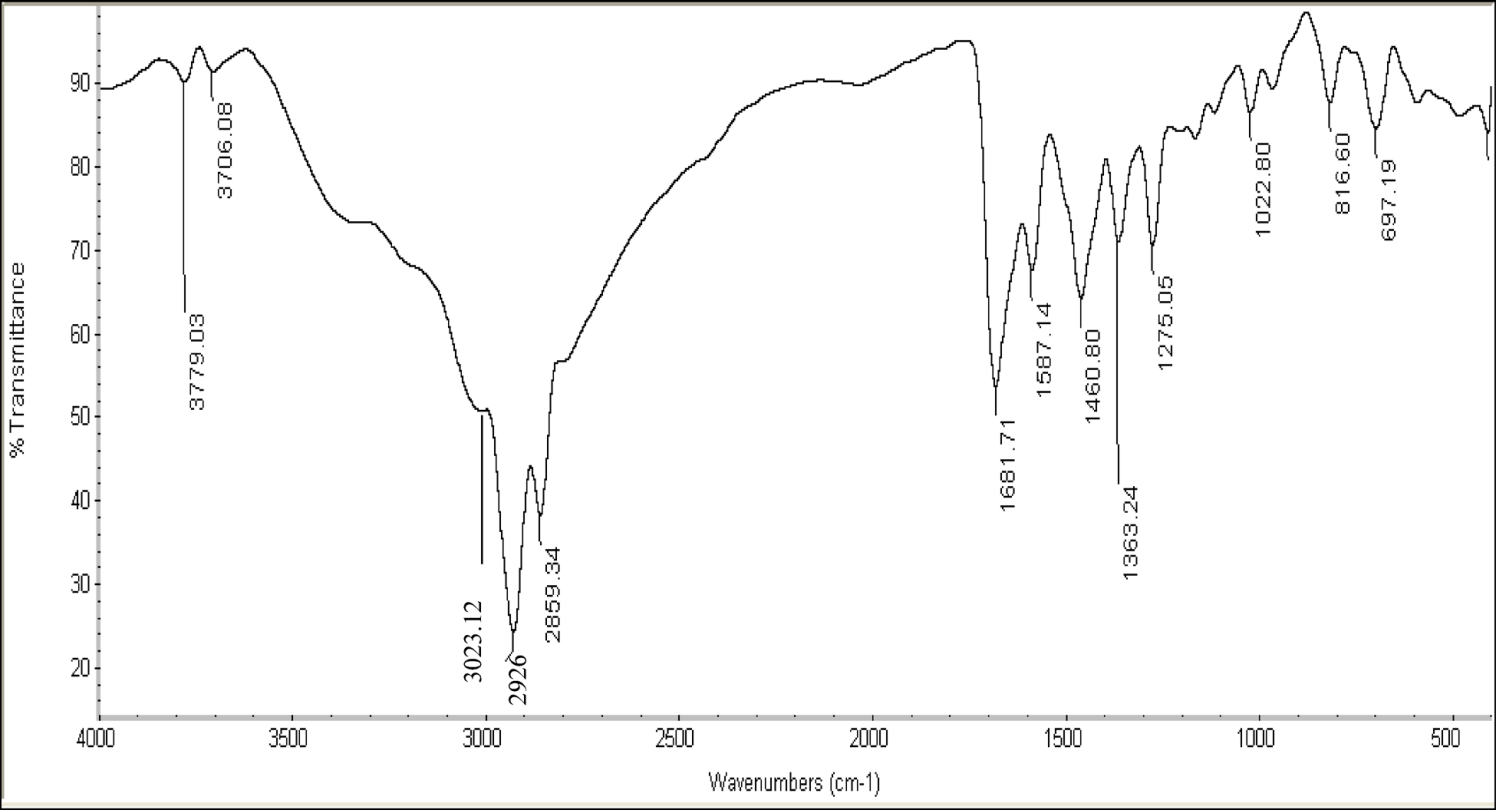
FTIR spectrum of the synthesized cationic gemini surfactants (IIIc).

**Fig. 3:**
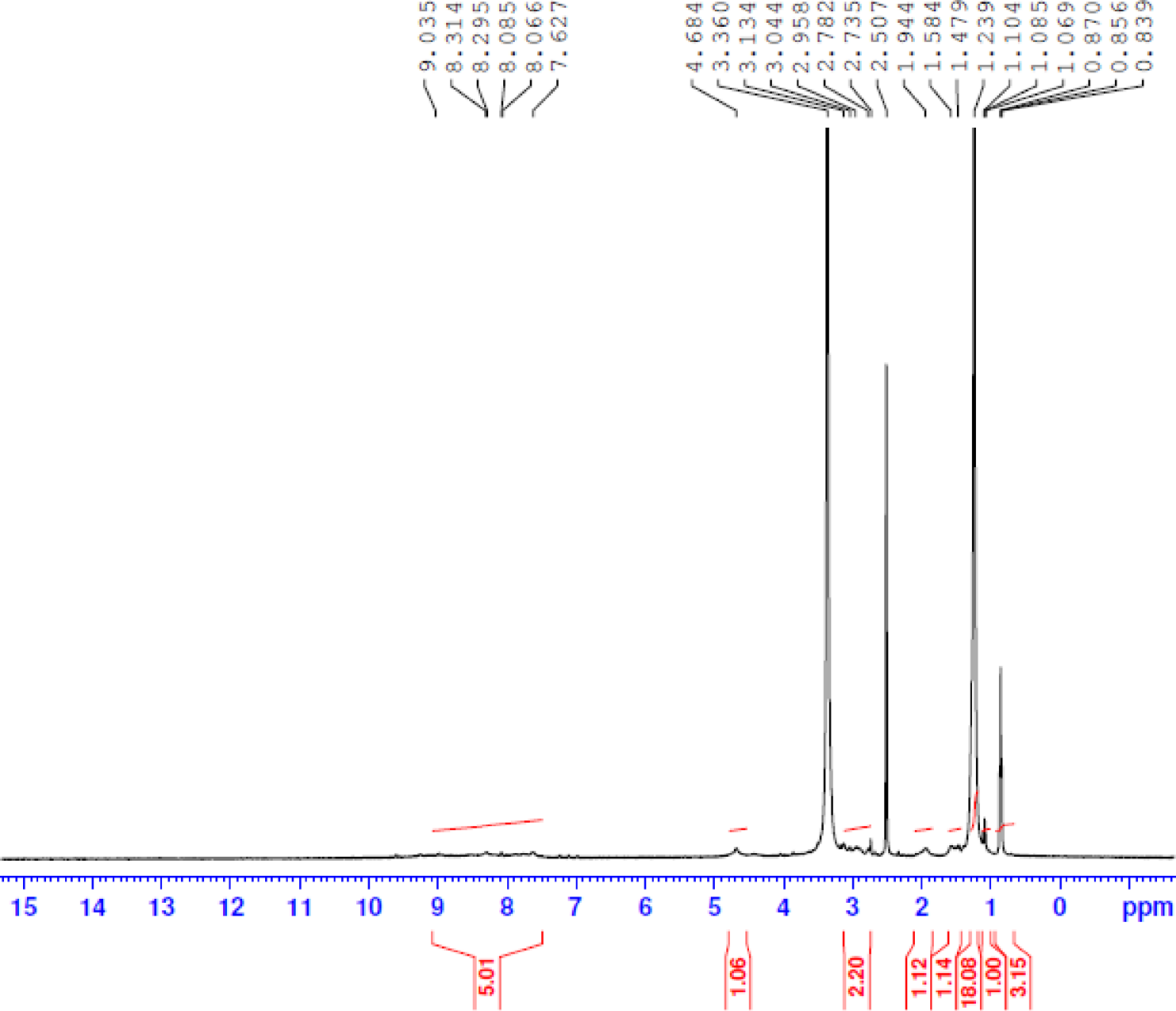
^1^H-NMR spectrum for synthesized cationic gemini surfactants (IIIc).

The synthesis of three cationic gemini inhibitor surfactants comprised three main steps, as depicted in Fig. 1^13^. The process involved:

(i) A Schiff base reaction between glyoxal (0.1 mol, 5.9 g) and ethylene diamine (0.2 mol, 12 g) via a condensation reaction in ethanol (the solvent) at 80 °C, stirred for 4 h^14^. After cooling, the solvent in the reaction mixture fully evaporated, yielding a dark yellow viscous product. This product was purified with diethyl ether and then recrystallized using ethanol^15^. The final product (I) was achieved with an 89.9% yield.
(ii) The Schiff base reaction of product (I) (0.1 mol, 2.28 g) with 3-acetyl pyridine (0.2 mol, 4.8 g) was conducted via a condensation reaction in ethanol at 80 °C, with stirring for 4 h to synthesize product (II) with an 88.7% yield.
(iii) The reaction of product (II) (1 mol, 1.89 g) with 2 moles of bromides (octyl C_8_, dodecyl C_12_, hexadecyl C_16_) was carried out through a quaternization reaction in absolute ethanol at 80 °C, with continuous stirring for 24 h^16^. After evaporating the solvent mixture to dryness, the resultant products were dissolved in methanol and extracted with hexane to remove any excess alkyl halide. This purification process yielded pure products IIIa, IIIb, and IIIc achieving approximately 87% yield., as shown in Fig. 1. The chemical structures of the three inhibitors were confirmed using FTIR and ^1^H NMR spectroscopy, as illustrated in Figs. 2 and 3.

### Gravimetric measurement

After abrasion with emery paper (grades 320–500–800–1000–1200), the carbon steel sheets, sized at 2.5 cm × 7.5 cm × 0.5 cm, underwent washing with distilled water and acetone. Subsequently, the weights were precisely measured, and the samples were placed in a lidded 150 ml beaker containing 100 ml of 1.0 M HCl, with and without inhibitors at varying concentrations. Following a 24-h period, the specimens were taken out, washed, dried, and accurately reweighed. Each experiment was triplicated, involving three parallel carbon steel sheets, and the.

average weight reduction was determined. The chemical composition of the carbon steel sample includes 0.19% C, 0.009% P, 0.004%S,0.05% Si, 0.94% Mn, 0.014% Ni, 0.009% Cr, 0.034% Al, 0.016% V, 0.003% Ti, and 0.022% Cu, with iron comprising the remaining composition.

### Electrochemical techniques

We recorded the electrochemical curves using an Origaflex potentiostat. The electrochemical cell described comprises an electrochemical cell with a working electrode (WE), a saturated calomel electrode (SCE) reference, and platinum counter electrode (CE). The carbon steel (CS) working electrode (WE) was set within PVC container with epoxy resin, revealing a 0.34 cm² of its surface to the solution. Before testing, the working electrode (WE) was placed in the test solution. and allowed to equilibrate at its open circuit potential (OCP) for 30 min. This pre-immersion period is crucial for achieving a stable state before conducting electrochemical measurements. Potentiodynamic Polarization (PP) measurements are a fundamental electrochemical technique used to assess the corrosion behavior of materials. The potential was swept from − 800 to − 300 mV relative to the Saturated Calomel Electrode (SCE). A scan rate oof 0.2 mV/s was used, with the temperature held at 20 °C.

### Surface analysis

Scanning Electron Microscopy (SEM) and Energy-Dispersive X-ray (EDX) spectroscopy became employed for the surface analysis of the carbon steel (CS) coupons. Samples, this analysis was performed on samples after their exposure to both blank and inhibited solutions (Zeiss Evo 10 Germany).

### Confirming the chemical structure of the synthesized cationic gemini inhibitor surfactants

Spectroscopic methods were employed to analyze the chemical structures of the synthesized cationic Gemini inhibitor surfactants (IIIa, IIIb, IIIc) are shown:

- “FTIR Spectroscopy was carried out on a Mattson ATI Genesis FTIR spectrometer at the Egyptian Petroleum Research Institute. Each spectrum was collected with a resolution of 2 cm^− 1^ and an 80° incidence angle.”
- We used Proton Nuclear Magnetic Resonance (^1^H NMR) spectroscopy to analyze the prepared compounds. These analyses were conducted in dimethyl sulfoxide on a Jeol ECA NMR spectrometer (Japan). The measurements were carried out at Faculty of Pharmacy - Ain Shams University.

### Surface-active parameters

Using the surface tension technique, the surface-active parameters for the solutions of cationic Gemini inhibitor surfactants (IIIa, IIIb, and IIIc) were measured at 25 °C. An Attention Theta Optical Tensiometer from Biolin Scientific Company (Finland) was employed to determine these values. An Attention Theta Optical Tensiometer (Biolin Scientific Company, Finland) was employed to determine these values. Subsequently, the surface parameters were calculated by averaging the measurements taken at each concentration. The concentration range used for determining the critical micelle concentration (CMC) of the Gemini surfactants was (0.1–0.000001 M/L). the concentrations were determined at which micelles form (CMC) values for the cationic Gemini inhibitor surfactants at a specified temperature by identifying the inflection point on the curve of the relationship between surface tension (γ) and the logarithm of surfactant concentration (logC). the effectiveness ((π_CMC_​)) of the cationic gemini inhibitor surfactants (IIIa, IIIb, and IIIc) was determined.

For each of the three synthesized cationic Gemini inhibitor surfactants. Additionally, the Gibbs adsorption equation was applied to determine the maximum surface excess (Γ_max_​), a value representing the greatest concentration attained at the solution interface per unit area^20^. The minimum surface areas (A_min_)) for the cationic gemini inhibitor surfactants (IIIa, IIIb, IIIc) were determined using the Gibbs adsorption equation^21^. Here, A_min_​ signifies value represents the average area each surfactant single unit occupies at the interface, with N being Avogadro’s number.

## Results and discussion inhibitors-structure characterization

Our research focused on synthesizing three cationic Gemini inhibitor surfactants, each with a different length of alkyl chain. Spectroscopic techniques confirmed the structures of the Gemini inhibitor surfactants produced.

### Confirmation of the cationic gemini inhibitors surfactants structure

#### FT-IR spectroscopy

The Fourier-Transform Infrared (FT-IR) spectrum of the prepared compounds showed bands at 697.19 cm^− 1^ (C-H bend for pyridine ring), 816.60 cm^− 1^ (= C-H bend), 1363.24 cm^− 1^ (CH_3_ symmetric bending), 1460.80 cm^− 1^ (CH_2_ asymmetric bending), 1681.71 cm^− 1^ (C = N), 2859.34 cm^− 1^ (CH symmetric stretching), 2926 cm^− 1^ (CH asymmetric stretching). After quaternization the FT-IR spectrum showed a characteristic absorption band for quaternary nitrogen at 3000–3035 cm^− 1^^19^. Confirmation of the cationic gemini inhibitor surfactants predicted functional groups was achieved through FT-IR spectroscopy Fig. 2.

#### ^1^H NMR spectroscopy

1 H NMR spectrum of the prepared compounds. The 1 H NMR ((DMSO)) spectrum exhibited different bands at δ = (0.856–0.870) ppm for [ NCH_2_CH_2_(CH2)_n_ *CH*_*3*_]; δ =(1.239) ppm for [*(CH*_*2*_*)n*-CH_3_] groups ; δ = (2.507) ppm for [*CH*_*2*_–N^+^] group after quaternization ; δ = (3.360) ppm for (N*CH*_*2*_*CH*_*2*_N); δ = (4.684) ppm for [N*CH*_*2*_ CH_2_(CH_2_)nCH_3_] ; δ = 8.066 ppm (m-pyridine); δ = 8.295 ppm (o-pyridine) ; δ = 9.035 ppm (− HC = N ).

The data on ^1^HNMR spectroscopy provided evidence for the distribution of hydrogen protons cationic gemini inhibitors Fig. 3.

### Surface active properties of the prepared cationic gemini surfactants

Parameters related to micellization, such as These parameters—critical micelle concentration (CMC), minimum surface area (A_min_​), maximum surface excess (Γ_max_​), and thermodynamic values—are crucial for various applications^22^. In this study, conventional methods were employed to analyze Aqueous surface properties of the cationic Gemini surfactants.

#### Critical micelle concentration (CMC) and (γ _CMC_)

The key characteristics of surfactants containing their propensity to accumulate at interfaces and form micelles. The critical micelle concentration ((CMC)) values for the synthesized compounds depend on the carbon chain length of their substituents.

**Fig. 4:**
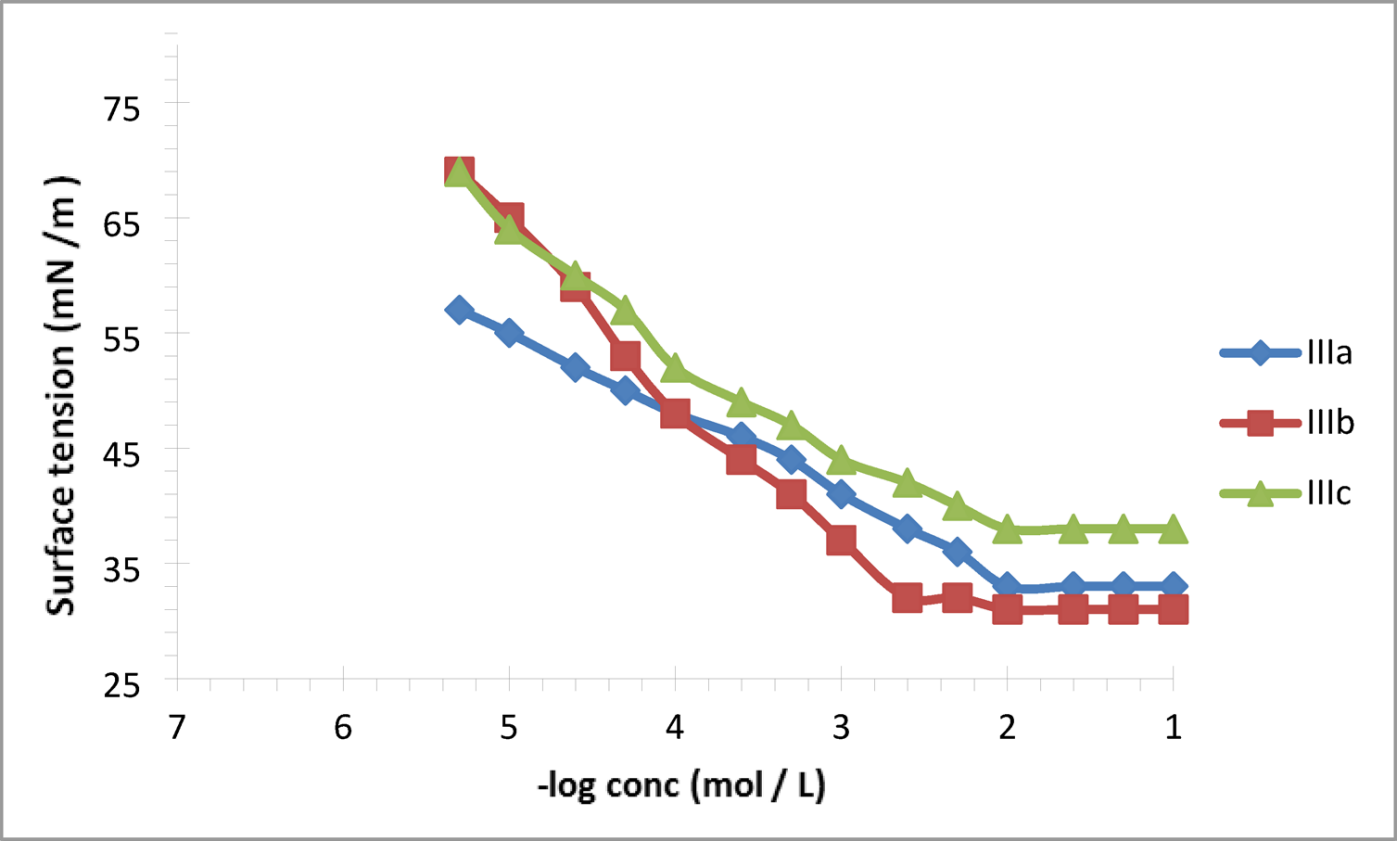
Surface tension variation with log concentration for synthesized cationic gemini surfactants (IIIa, IIIb, IIIc) at 25 °C.

Plots of surface tension ((γ)) versus the logarithm of the concentration for the prepared cationic gemini inhibitor surfactants IIIa, IIIb, and IIIc as seen in Fig. 4. The point where the curves change could be linked to critical micelle concentrations. The critical micelle concentration (CMC) is the lowest concentration at which surfactant molecules begin to clump together and form micelles. Values of CMC and γcmc of synthesized cationic gemini inhibitor surfactants IIIa, IIIb and IIIc are collected in Table 1.

The data in Table 1 indicates that, all the investigated cationic gemini surfactants IIIa, IIIb and IIIc showed very low CMC values for micellization in water. his means cationic Gemini inhibitor surfactants are more likely to self-assemble at lower concentrations in water.

As shown in Table 1, the length of the surfactant’s hydrophobic tail plays a crucial role. Extending hydrophobic tails (from C8​ to C12​) led to lower measured CMC values (Table 1 and Fig. 4). The carbon chain length of surfactants significantly affects their behavior at the interface between liquid and air. As the carbon chain increases, surfactant molecules migrate more readily to the interface, where they quickly form a monolayer. This initial coverage facilitates the subsequent formation of micelles. Longer carbon chains enhance micelle formation, making it thermodynamically favorable^23^.

Interestingly, our observations indicate Instead of decreasing as expected, increasing the hydrophobic tail length from C12​ to C16​ actually raised the CMC values (see Fig. 4 and Table 1). In case of (C16), there was an increase in CMC value, which may be interpreted by the occurrence of self-coiling of the surfactant molecules, which generally appears only when the carbon atoms in hydrophobic chains are more than 14 carbon atoms^23^. Also, this counterintuitive result may stem from the increased steric hinderance and hydrophobicity caused by the two long alkyl chains of C16, in conjunction with two pyridine rings^16,21^.

Generally, the cationic gemini that were synthesized inhibitor surfactants exhibit remarkable surface-active and Self-aggregation behavior in water. Specifically, The CMC values for the cationic Gemini surfactants (IIIa, IIIb, and IIIc) were calculated at 25∘C and are presented in Table 1. Surfactant IIIb had the optimal CMC value. The critical micelle concentration ((CMC)) of the cationic gemini inhibitor surfactants was observed to change based on the hydrophobic tail length of the prepared surfactants.

Table 1 presents the surface tension values at the critical micelle concentrations ((γ_CMC_​)) for each of the synthesized cationic gemini inhibitor compounds. CMC results showed that surfactant IIIb had a lower surface tension ((γ_CMC_​)) than the other surfactants.

#### Effectiveness (π_cmc_)

The equation below allows us to calculate the maximum surface pressure (πCMC​), a measure of how effectively a surfactant lowers surface tension.

1 $${\pi}_{cmc}={\gamma}_{o}-{\gamma}_{cmc}$$

where γ _o_​ represents the surface tension of pure water and γ _cmc_ ​ is the surface tension at the critical micelle concentration^24^. Efficacy values are a key benchmark for comparing the effectiveness of two surfactants within the same series. We identify the most effective surfactant by its lowest surface tension at the critical micelle concentration ((CMC)). As indicated in Table 1, IIIb was the most effective at lowering surface tension at its critical micelle concentration ((CMC)).

#### Maximum surface excess (Γ _max_)

A key factor in assessing Surfactant surface activity is determined by the packing efficiency of surfactant molecules at the air-water interface^25^. We calculated the maximum surface excess concentration (Γ_max_​) of surfactant ions at this interface through the use of the appropriate Gibbs adsorption equation. based on the linear slope of the surface tension plot (dγ/dlog c) below the critical micelle concentration (CMC).

2 $${{\Gamma}}_{max}=-{({{\delta \gamma}/{\delta logc})}_{T}}/{2.303nRT}$$

In this equation, Γ_max_​ is the maximum surface excess concentration of surfactant ions, R is the gas constant, T is the absolute temperature, and ((dγ/dlogc)) is the slope observed in the plot of surface tension (γ) against the logarithm of concentration (log c)^26^.

The prefactor ‘n’ represents the number of species present at the air-water interface. For gemini surfactants, n is typically considered to be 3, reflecting the presence of a divalent surfactant ion.

The effectiveness of surfactants at various interfaces relies on their adsorption capacity. The differences in surface activity at the air/water interface among various surfactants arise from variations in their packing density, represented by Γ_max_ and A_min_​. As the surface excess concentration increases, the surface area value tends to decrease^27^.

Values of Γ_max_, as shown in Table 1, indicate that the slope of the surface tension plot dγ/dlogc effects on the highest surface excess achieved.

#### Minimum surface area (A_min_)

The area per molecule at the interface reveals how densely packed and oriented the adsorbed surfactant molecules. Amin​: The average minimum surface area (in Å²) the interfacial area per adsorbed molecule^28^ is calculated as:

3 $${A}_{min}={{10}^{16}}/{{N}_{A}{{\Gamma}}_{max}}$$

Where N_A_​ is Avogadro’s number and Γ_max_​ (mol m^− 2^) is the maximum surface excess of surfactant molecules adsorbed at the interface. When Γ_max_​ decreases, it means there are fewer molecules at the air/water interface. Consequently, the data in Amin​ illustrates that each molecule will have more area available at the interface (Table 1).

#### Standard free energies of micellization and adsorption (ΔG_mic,_ ΔG_ads_)

Adsorption and micellization are essentially phase transformation processes, as surfactants transition from individual molecules in solution to organized structures, adsorbed molecules at the interface, or as micellar aggregates (in the case of micellization).

We calculated the standard free energies of micellization and adsorption (ΔG^°^_mic_​ and ΔG^o^_ads_​) using the Gibbs adsorption rules^29^ as follows:

4 $${\Delta}{G}_{mic}^{o}=nRT\mathrm{ln}CMC$$

5 $${\Delta}{G}_{ads}^{O}={\Delta}{\mathrm{G}}_{mic}^{o}-6.023\times{\pi}_{cmc}\times{A}_{min}$$

where R is the gas constant, T is the absolute temperature, and CMC is represented as the surfactant’s molarity. According to Table 1, The negative ΔG^o^ mic​ and ΔG^o^ads ​ values for these cationic Gemini inhibitor surfactants show that both adsorption and micellization happen without external intervention. This reduced the rise in solution energy caused by the solubility of amphiphilic compounds in water. The information presented in Table 1 indicates that for the synthesized cationic Gemini surfactants at 25 °C, compared to the ΔG_mic_∘​ values, the ΔG_ads_^o^​ values are more negative. In an aqueous solution, IIIa, IIIb, and IIIc first adsorb at the solution interface, reaching a saturated state, after which they aggregate to form micelle structures.

**Table 1:** Critical micelle concentration (CMC) and surface parameters for cationic gemini surfactants IIIC8, IIIC12, and IIIC16 at 25 °C.

Surfactant	CMC (mmol / l)	γ_CMC_ (mN/m)	πcmc (mN/m)	(Γ_max_×10^11^ (mol/cm^2^)	A_min_ (nm^2^)	ΔG^o^_mic_ kJ/mol	ΔG^o^_ads_ kJ/mol
(IIIC_8_)	0.237	34.57	37.43	3.59	4.62	– 20.68	– 20.80
(IIIC_12_)	0.169	31.52	40.48	5.38	3.09	– 21.51	– 21.60
(IIIC_16_)	0.305	39.35	32.65	4.26	3.90	– 20.06	– 20.10

### EIS results

**Fig. 5:**
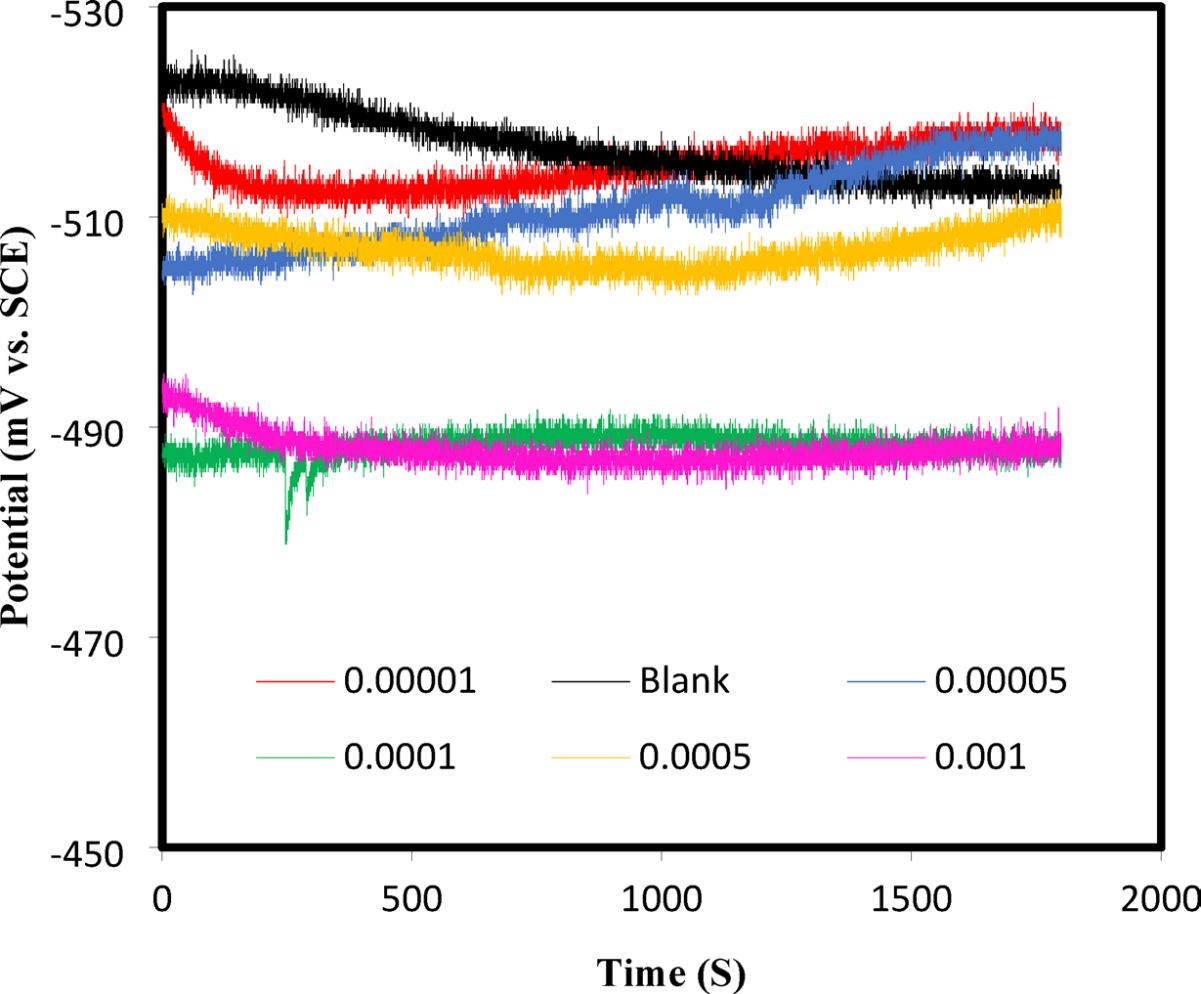
Open circuit potential (OCP) for carbon steel in 1 M HCl in absence and presence of the inhibitor IIIb.

**Fig. 6:**
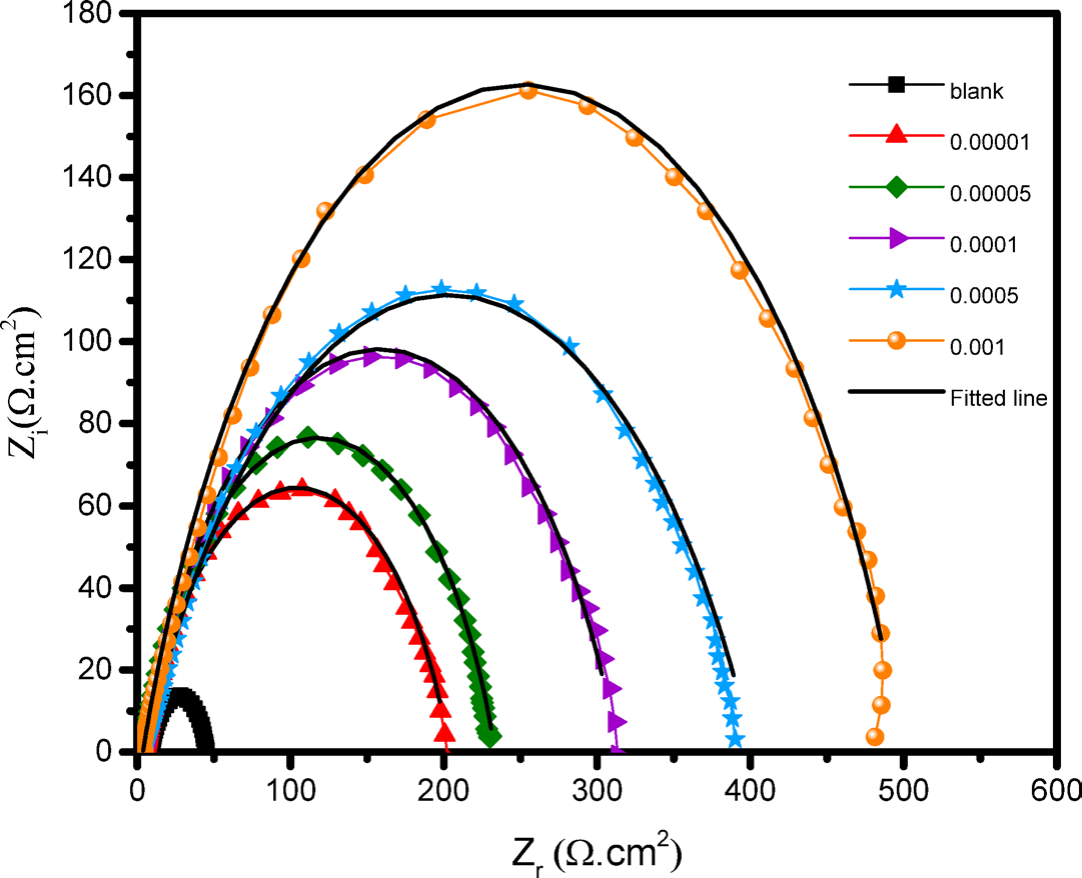
Nyquist plots for CS at 25 °C in 1.0 M HCl with and without different concentrations of III_b_ of CGIS.

In this work, the mechanism of inhibition of the CGIS IIIa, IIIb, and IIIc on the CS surface in 1.0 M HCl at room temperature is revealed by the electrochemical impedance spectroscopy (EIS) technology. Open circuit potential (OPC) for carbon steel in 1 M HCl in absence and presence of the inhibitor IIIb was shown in Fig. 5. The semicircular capacitive loops of CS in 1.0 M HCl solution are shown in Fig. 6 with and without different IIIb concentrations. Under typical circumstances, frequency dispersion—which results from surface heterogeneity or the relaxation dynamics of intermediate species—causes a departure from the ideal capacitive response. The CS surface is uneven and inhomogeneous, which results in frequency scattering in the Nyquist plot^30^. As CGIS concentrations are increased, a supplement to the CGIS film that develops on the CS surface causing an increase in charge transfer resistance (R_ct_)^31^.

**Fig. 7:**
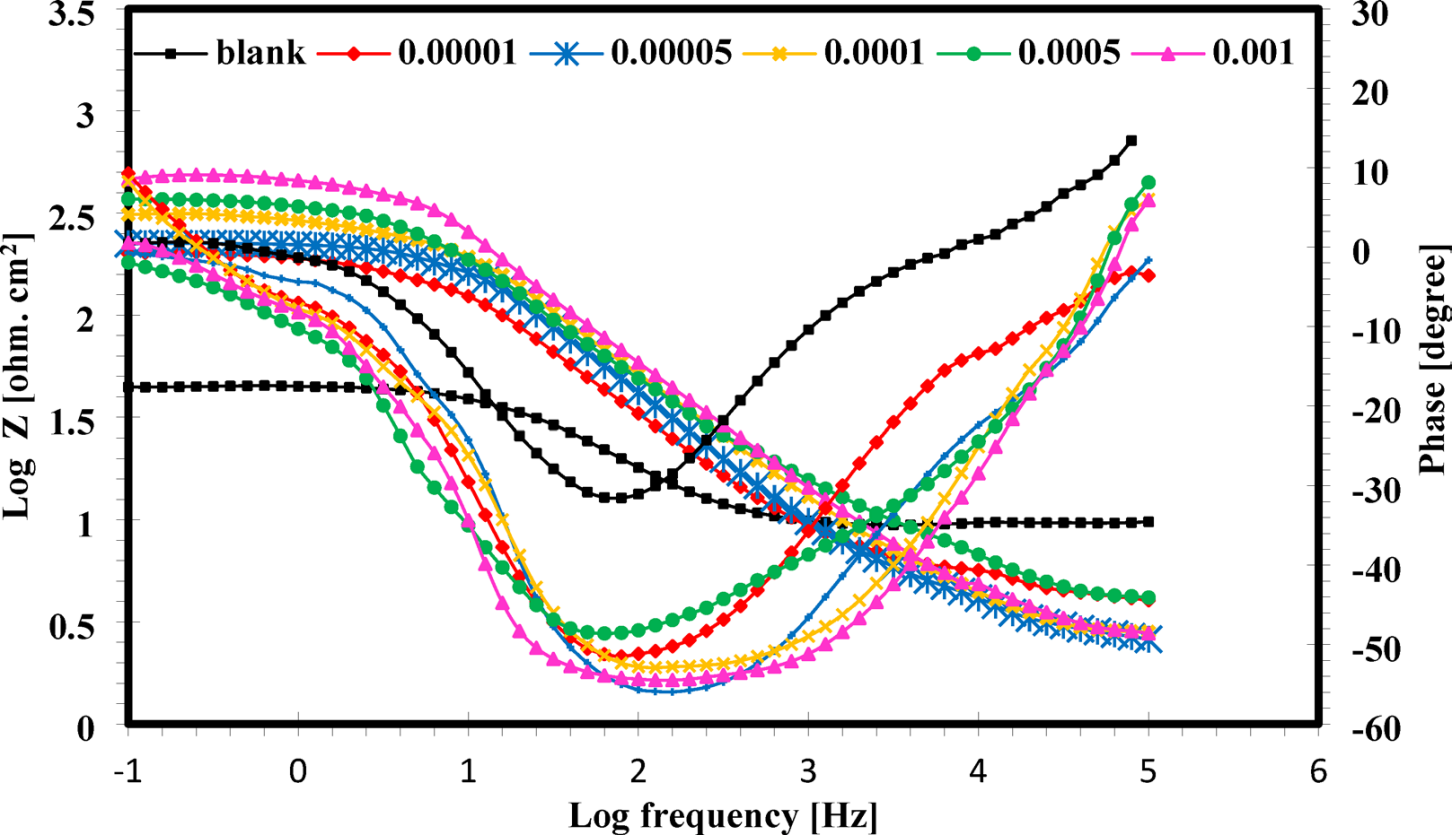
Bode and phase plots for CS at 25 °C in 1.0 M HCl with and without different concentrations of III_b_ of CGIS.

**Fig. 8:**
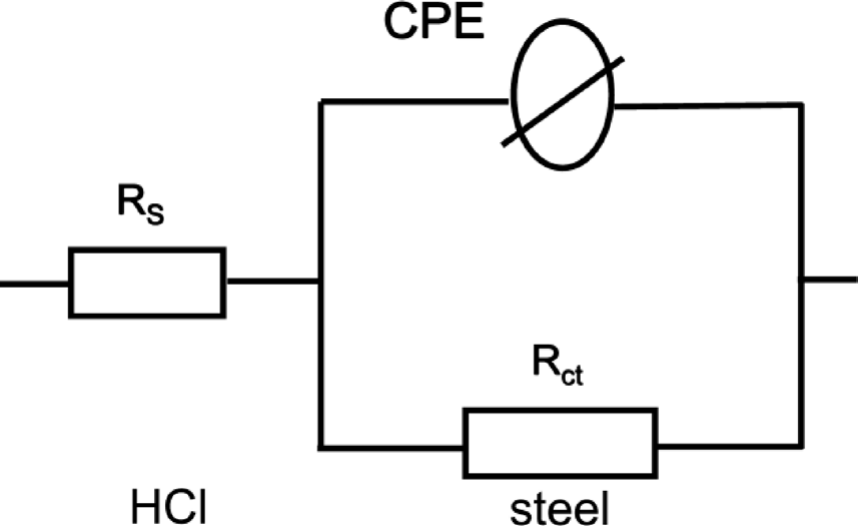
The Equivalent circuit for the fitting the impedance measurements data.

The nyquist plots show that these inhibitors do not change the inhibitory mechanism, maintaining the same electrochemical behavior as the blank solution, both with and without the addition of CGIS^32^. The bode plot in Fig. 7 shows only one time constant, which graphs the phase angle against frequency. This indicates that this system’s equivalent circuit is simplified circuit. The equivalent circuit used for fitting the EIS spectra consists of the charge transfer resistance (Rct) parallel to the constant phase element (CPE) and the solution resistance (Rs) lies in series Fig. 8. An electrical capacitor is involved in a double layer between the charged metal surface and the solution. Because of the imprecise semicircle form of Nyquist plots, CPE replaced the double layer capacitance (Cdl). CPE impedance (ZCPE) is calculated using the formula below^33^:

6 $${Z}_{CPE}={Y}_{0}^{-1}{\left(i{\omega}_{max}\right)}^{-n}$$

7 $$\omega=2\pi{f}_{max}$$

The constant phase element, angular frequency, frequency at the maximum imaginary element of the impedance, imaginary number, and coefficient of surface inhomogeneity are represented by the symbols Y_o_, ω_max_, f_max_, i, and n, respectively. The following procedure is used to determine the η_I_^34,35^:

8 $${\eta}_{I}=\frac{{R}_{ct}^{o}-{R}_{ct}}{{R}_{ct}^{o}}$$

where the charge-transfer resistances with and without the inhibitor are denoted by R_ct_ and R°_ct_, respectively.

The electrochemical parameters derived from EIS Table 2 verify that R_ct_, which is dependent on CGIS concentration, determines inhibitory efficiency. Furthermore, it is clear that the CGIS’s n values, which are lower than those of the blank solution and show the usual non-ideal capacitive activities, range from 0.64 to 0.78. By comparing the values of CPE for CS, the blank HCl solution is higher than the inhibited CGIS solution, the in. The η_I_ values for CGIS increase with the R_ct_ values. It is evident that IIIb exhibits the highest inhibitory efficacy.

**Table 2:** Impedence parameters for carbon steel without and with different doses of the synthesized inhibitors III _a_, III _b_ and III _c_ in 1.0 M HCl.

Inhibitors	Conc. of inhibitors (mol/L)	*R*_s_ (Ωcm^2^)	CPE, Y_o_ × 10^− 4^ (µΩ s^*n*^ cm^− 2^)	*n*	*R*_ct_ (Ωcm^2^)	η_I_%
Blank	-----	9.41	3.66	0.83	35.71	----
III_a_	1 × 10^− 5^	9.13	2.04	0.70	179.1	80.06
	5 × 10^− 5^	4.61	2.60	0.72	217.6	83.59
	1 × 10^− 4^	6.62	2.40	0.68	294.4	87.87
	5 × 10^− 4^	4.81	2.035	0.65	356.1	89.97
	1 × 10^− 3^	5.42	2.73	0.64	438.8	91.86
III_b_	1 × 10^− 5^	4.60	2.60	0.73	199.3	82.02
	5 × 10^− 5^	2.92	1.69	0.75	229.2	84.42
	1 × 10^− 4^	2.91	1.70	0.71	310.8	88.51
	5 × 10^− 4^	4.38	2.703	0.65	397.5	91.01
	1 × 10^− 3^	3.73	1.30	0.74	495.4	92.79
III_c_	1 × 10^− 5^	2.07	2.36	0.76	175.8	79.68
	5 × 10^− 5^	2.58	2.10	0.78	221.0	83.84
	1 × 10^− 4^	4.21	1.67	0.76	294.3	87.86
	5 × 10^− 4^	4.56	2.31	0.71	355.9	89.96
	1 × 10^− 3^	3.40	1.68	0.71	436.2	91.81

#### Potentiodynamic polarization measurements

**Fig. 9:**
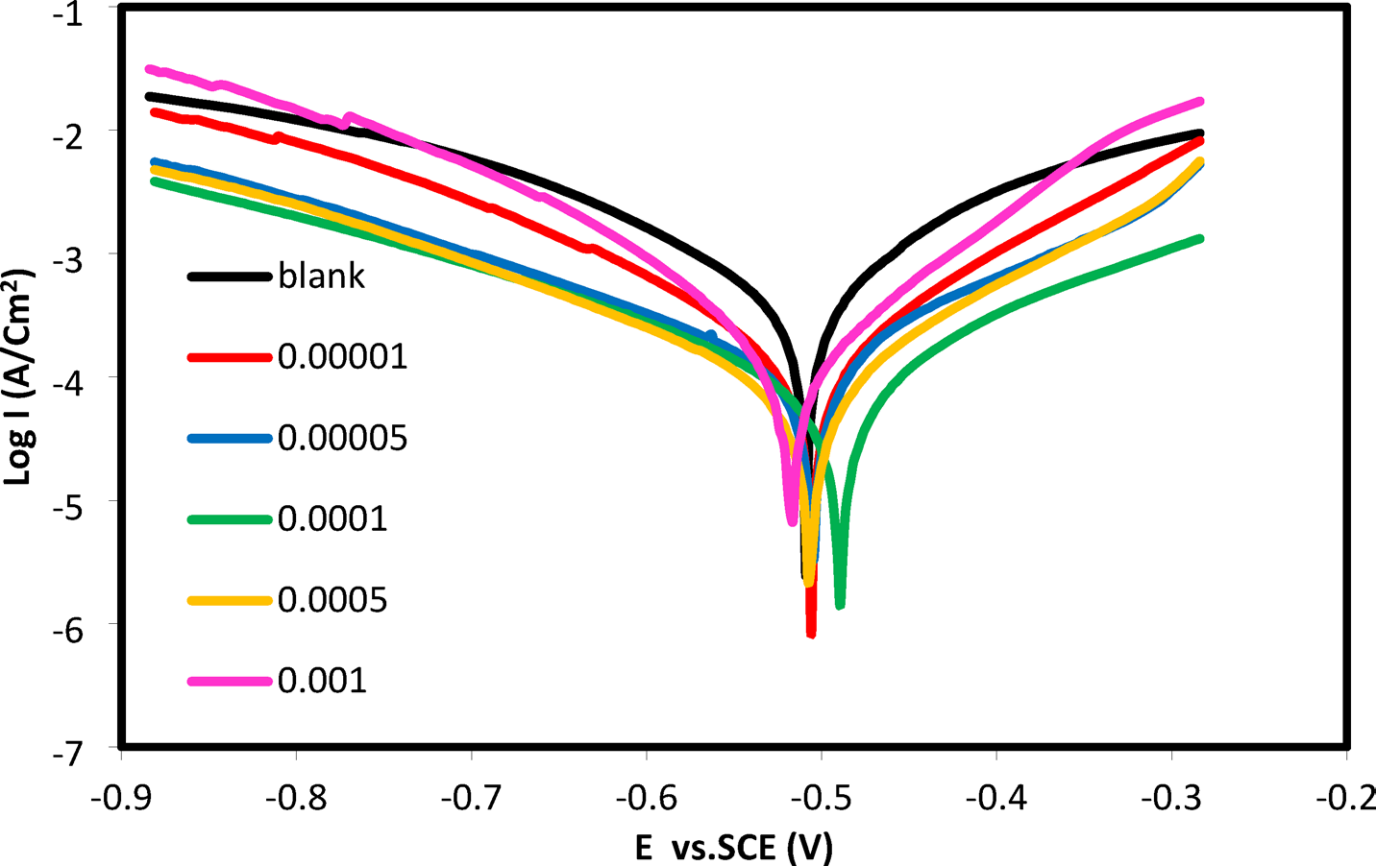
Anodic and cathodic polarization curves for CS at 20 °C in 1.0 M HCl with and without different concentrations of IIIb of CGIS.

Figure 9 showed the CGIS’s IIIb concentrations at 25 °C as well as its functionality. When compared to the blank curve, it is clear that the addition of CGIS moved the CS’s I-V curves to a more noble potential. This change illustrates the inhibitors’ protective action, as they contain functional groups such as benzene rings possessing π electrons, quaternary nitrogen and azomethine group (-N = CH-) which are high adsorption centers^32^. These organizations strengthen the CS surface’s resilience to corrosion by encouraging chemical and physical interactions with it. The electrochemical corrosion parameters obtained from the tafel line extrapolation were given in Table 3. The degree of surface covering (θ) and corrosion inhibition efficiency (IE%) of the produced CGIS were calculated using the following formulas^36–38^:

9 $${\eta }_{p}\%=\left(\theta\times 100\right)=\frac{{i}_{corr}^{o}-{i}_{corr}}{{i}_{corr}^{o}}\times 100$$

where i_corr_ and i^o^_corr_ stand for corrosion current densities when different concentrations of CGIS IIIa, IIIb, and IIIc are present and absent, respectively. As the concentration of the CGIS inhibitor rises, the i_corr_ values fall, indicating more adsorption of these inhibitors on the CS surface^39^. The CS surface adsorbs more molecules as the concentration of CGIS inhibitors increases, facilitating the electrostatic interaction between the surfactant’s quaternary ammonium cation (N^+^) and the chloride anions (Cl^−^) that are already adsorbed on the CS surface.

This improved electrostatic contact reinforced the donor-acceptor link between the active centers of the surfactant and the vacant d orbitals of the carbon steel. The enhanced corrosion inhibition efficiency (η_p_%) and increased surface coverage (θ) with increasing CGIS concentration demonstrate that the CGIS inhibitor molecules formed a stable adsorbed film^40^.

IIIb CGIS has the highest corrosion inhibition efficiency as opposed to the blank sample of CS. The corrosion potential (E_corr_) reduced by less than 85 mV when CGIS inhibitors were present^40–42^, suggesting that these inhibitors are classified as mixed-type inhibitors, which lower both anodic and cathodic processes. By slowing both the anodic metal dissolution and cathodic oxygen reduction processes, the addition of CGIS inhibitors appears to have had no effect on the mechanism of the anodic and cathodic reactions, based on the slight change in tafel slopes (βa and βc)^33,43^.

**Table 3:** Potentiodynamic polarization parameters for carbon steel without and with different concentrations of CGIS in 1.0 M HCl.

Inhibitors	Conc. of inhibitors (mol/L)	- E_corr_. (mV)	i_corr_. (mA/cm2)	β_a_ (mV/dec)	- β_c_ (mV/dec)	η_*p*_%
Blank	–	509.0	0.8636	191	230.9	–
IIIa	1 × 10^–5^	462.9	0.1478	157.7	173.5	82.88
	5 × 10^–5^	501.7	0.1222	148.6	167	85.85
	1 × 10^–4^	460.9	0.0989	115.9	174.4	88.55
	5 × 10^–4^	487	0.0772	157.6	184.7	91.06
	1 × 10^–3^	470	0.0662	107	172.9	92.33
IIIb	1 × 10^–5^	505.7	0.1394	125.1	138.9	83.85
	5 × 10^–5^	504.7	0.1177	149.5	208.6	86.37
	1 × 10^–4^	489.6	0.0888	159.8	216.7	89.71
	5 × 10^–4^	507.2	0.0638	122	156	92.61
	1 × 10^–3^	488.6	0.0568	113	168.2	93.42
IIIc	1 × 10^–5^	513.3	0.1499	141.2	161.8	82.64
	5 × 10^–5^	517.2	0.1228	136.1	162.3	85.78
	1 × 10^–4^	505	0.1035	144.2	197.9	88.01
	5 × 10^–4^	469	0.0865	113.4	192.5	89.98
	1 × 10^–3^	486	0.0739	120.4	184.6	91.44

### Weight loss results

Equations (6) and (8) were used to determine the CGIS dissolving rate (k), surface coverage (θ), and anticorrosion efficiency (η_w_) in blank 1.0 M HCl and with CGIS -molecule:^44–46^

10 $$k=\frac{\varDelta W}{S \times t}$$

11 $$\theta=\frac{({W}_{un}-{W}_{in})}{{W}_{un}}$$

12 $${\eta}_{w}=\frac{\left({k}_{un}-{k}_{inh}\right)}{{k}_{un}}\times100$$

S is the CS-surface area in cm^2^, k_un_ and k_in_ stand for the rates of corrosion for the blank solution 1.0 M HCl alone and for solutions with the CGIS-molecule. In both the blank 1.0 M HCl and the CGIS-molecule, W_un_ and W_in_ represent the mass loss of CS, and the mean weight loss (WL) of CS-coupons is denoted by ∆W.

Table 4 shows the weight loss properties of CS at different CGIS concentrations and in blank 1.0 M HCl. This surfactant successfully prevents CS from corroding since it contains heteroatoms such N atoms. The degree of inhibition depends on the kind and manner of CGIS molecule’s adsorption onto the steel surface. According to WL studies, CGIS’s anticorrosion effectiveness rises with concentration^36,47,48^.

**Fig. 10:**
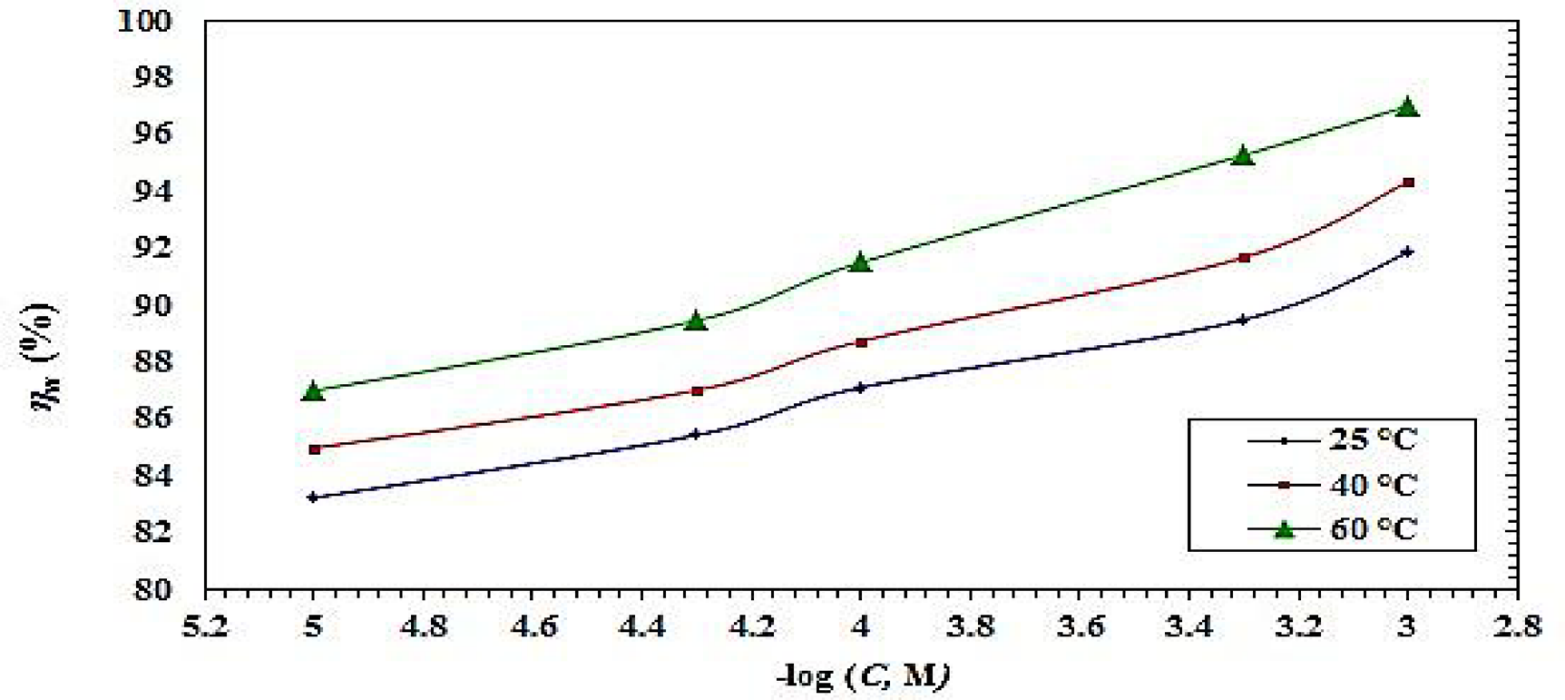
influence of temperature on η_w_ from weight loss method for CS in 1.0 M HCl alone and with various doses of IIIb of CGIS molecule.

Figure 10 shows how temperature affects η_w_ derived from weight loss (WL) investigations in the 25–60 °C temperature range for CS in 1.0 M HCl solution alone and for that with varying amounts of CGIS molecules. Table 4 displays the weight loss and corrosion rate data at various temperatures. These results show that the CS-surfaces corrode more rapidly with increasing temperature^49^.

The slight increase in η_w_ for CGIS with temperature indicating that these compounds are chemically adsorbed on CS surface^32,50^. The chemisorption process is promoted by the electron-rich functional groups that create strong contacts with Fe CS surface, such as diazomethine, SP^2^carbon, and nitrogen^32,51^. Chemical reactions at higher temperatures lead to increased electron densities at the adsorption centers, which strengthens the inhibitors’ adsorption on the coupon surface and raises η_w_. This interaction leads to the formation of a stable coordinate-type bond, creating a dense protective film that hinders the corrosion process., such chemical interaction is often supported by high negative values of Gibbs free energy and the stability of the inhibitor film at elevated temperatures, which is characteristic of the CGIS -based and similar organic inhibitors in aggressive acidic environments^52^.

**Table 4:** Weight loss and activation energy results for CS in 1.0 M HCl with and without different concentrations of CGIS at various temperatures.

Inhibitor name		25 °C			40 ^o^C			60 °C		
	Inhibitor conc. M	k mg cm^−2^h^− 1^	θ	ηw %	k mg cm^−2^h^− 1^	θ	ηw %	k mg cm^−2^h^− 1^	θ	ηw %
Absence		0.32127			0.899			3.1342		
IIIa	0.00001	0.0640	0.80	80.07	0.1677	0.81	81.35	0.5253	0.83	83.24
	0.00005	0.0556	0.83	82.70	0.1412	0.84	84.30	0.4325	0.86	86.20
	0.0001	0.0497	0.85	84.53	0.1248	0.86	86.12	0.3561	0.89	88.64
	0.0005	0.0394	0.88	87.73	0.0906	0.90	89.93	0.2294	0.93	92.68
	0.001	0.0279	0.91	91.30	0.0716	0.92	92.04	0.1670	0.95	94.67
IIIb	0.00001	0.054	0.83	83.22	0.135	0.85	84.96	0.403	0.87	87.14
	0.00005	0.047	0.85	85.41	0.1170	0.86	86.99	0.327	0.90	89.57
	0.0001	0.041	0.87	87.09	0.101	0.89	88.73	0.264	0.92	91.59
	0.0005	0.034	0.89	89.47	0.075	0.92	91.66	0.147	0.95	95.32
	0.001	0.026	0.92	91.85	0.051	0.94	94.33	0.093	0.97	97.03
IIIc	0.00001	0.070	0.78	78.06	0.189	0.79	79.03	0.548	0.83	82.52
	0.00005	0.059	0.82	81.57	0.153	0.83	82.97	0.454	0.86	85.52
	0.0001	0.054	0.83	83.25	0.135	0.85	84.94	0.401	0.87	87.19
	0.0005	0.041	0.87	87.21	0.100	0.89	88.93	0.301	0.90	90.40
	0.001	0.031	0.90	90.30	0.082	0.91	90.90	0.218	0.93	93.04

#### **Kinetics parameters**

The following describes the way the values of (*E*_a_) activation energy was calculated using Arrhenius relation^53^:

13 $$\mathrm{ln}k-\mathrm{ln}A=\frac{-{E}_{a}}{R\times T}$$

Where, A is the frequency index, R is the universal gas constant, and k is the CS’s corrosion rate.

**Fig. 11:**
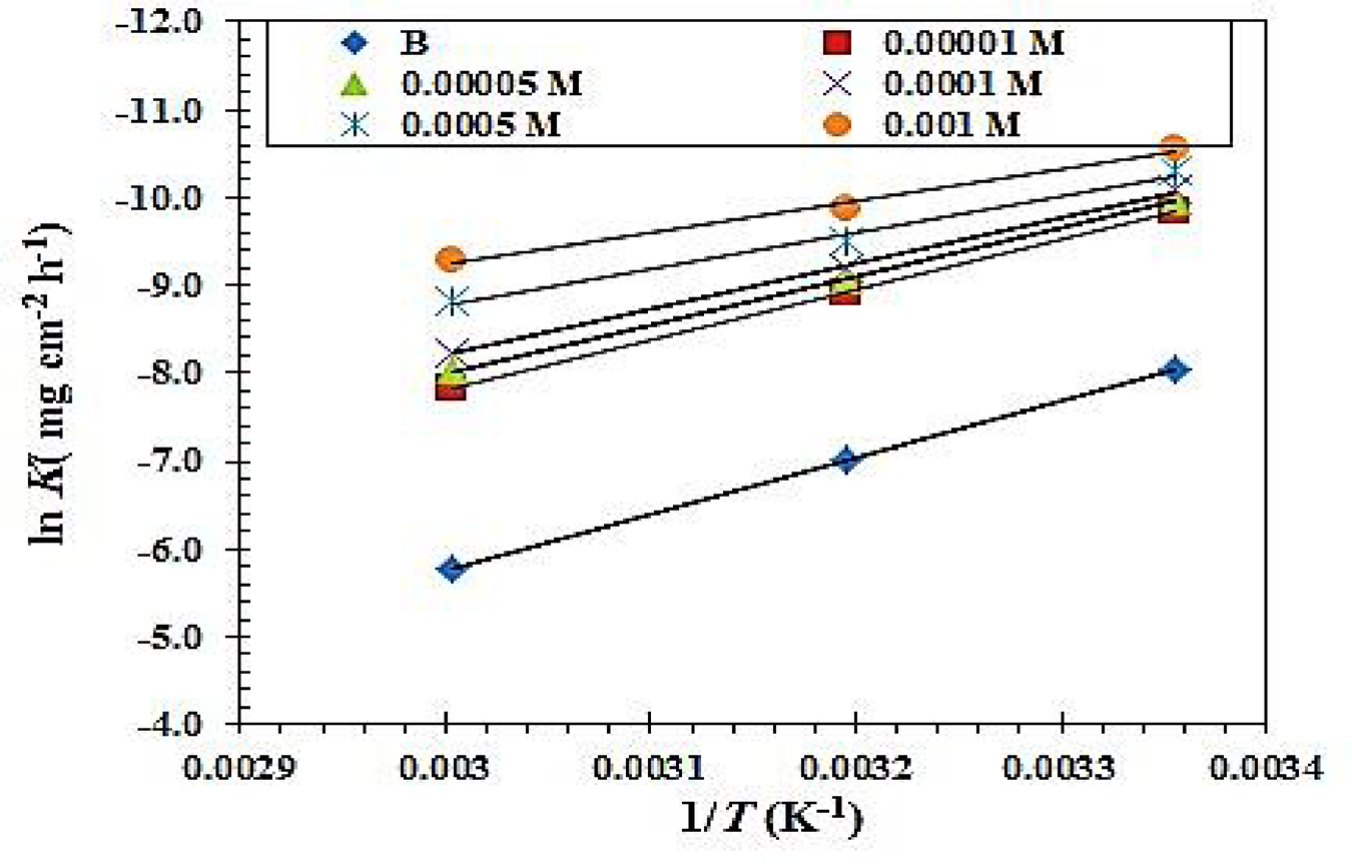
(ln *k* versus 1/T) curves for CS in 1 M HCl solution and with various doses of IIIb of CGIS molecule.

The association between (ln k and 1000/T) in a blank 1.0 M HCl solution and at various doses of the CGIS inhibitors under investigation for CS is shown in Fig. 11. Gained straight lines have a slope of (-Ea/R). Table 5 displays the results of the calculation of the *E*_a_ values. It should be mentioned that, in comparison to free 1.0 M HCl, the *E*_a_ values are reduced or somewhat altered when the CGIS molecule is present. Its chemical adsorption on the CS surface is the cause of this, whereas physical adsorption has the reverse effect^54^. The activation enthalpy (∆H*) and entropy (∆S*) were computed using the following transition state formula^53,55^:

14 $$\mathrm{ln}\left(\frac{k}{T}\right)+\frac{\varDelta{H}^{*}}{R\times T}=\left[\mathrm{ln}\left(\frac{R}{{N}_{A}\times h}\right)+\left(\frac{\varDelta{S}^{*}}{R}\right)\right]$$

where h and N are the Planck constant and Avogadro’s number, respectively.

**Fig. 12:**
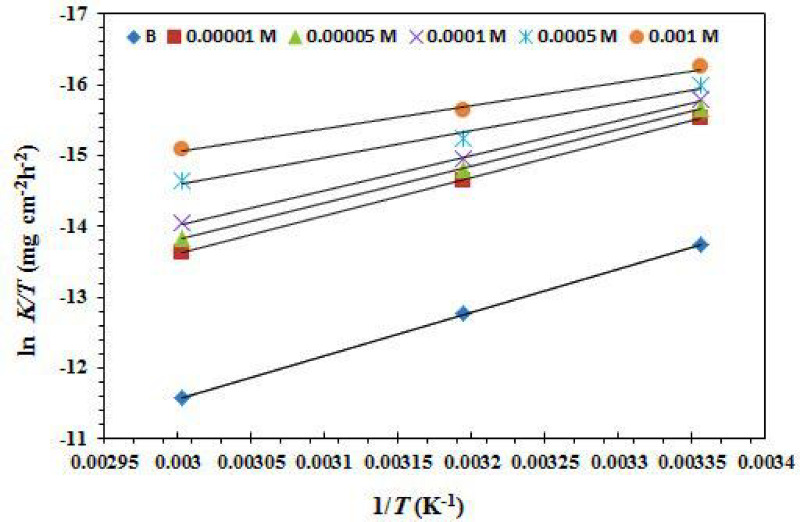
(ln *k*/T versus 1/T) relation for CS in the uninhibited 1.0 M HCl solution and the inhibited with IIIb.

For CS dissolution in the blank solution of 1.0 M HCl and with varying doses of (0.00001, 0.00005, 0.0001, 0.0005, and 0.001) M of CGIS molecule, Fig. 12 illustrates the relationship between ln (k×T^− 1^) and (1/T). The resulting straight lines have a slope of (-∆H*/R) and an intercept of ln [(R × N^− 1^ × h^− 1^) + ∆S* ×R^− 1^]. Table 5 shows the values of the two parameters, ∆H^*^ and ∆S^*^. The positive sign of ∆H* calculated values in both inhibitory and uninhibited solutions indicates that CS corrosion is endothermic^56^. Additionally, as the activated complex in the rate determination step displays a fusion at ions rather than disengagement, the negative values of ∆S* Table 5 suggest a reduction disruption during the transition from reactants to the activated complex. The values of ∆S* changed to more negative values as the CGIS concentration increased, showing a more stable pattern that enhanced the anticorrosion efficacy.

**Table 5:** Values of activation parameters for CS in uninhibited 1.0 M HCl and inhibited with various concentrations CGIS.

	Inhibitor conc. (M)	E_a_ (kJ mol^− 1^)	∆ H*_ads_ (kJ mol^− 1^)	∆S*_ads_ (J mol ^−1^K^− 1^)
B	0.0	53 ± 2.1	59.541	– 0.083
IIIa	0.00001	50 ± 2.1	46.950	– 0.110
	0.00005	48 ± 2.1	45.710	– 0.116
	0.0001	46 ± 2.1	43.727	– 0.123
	0.0005	41 ± 2.1	38.829	– 0.141
	0.001	42 ± 2.1	39.314	– 0.138
IIIb	0.00001	47 ± 2.1	44.748	– 0.119
	0.00005	34 ± 2.1	43.081	– 0.126
	0.0001	43 ± 2.1	40.871	– 0.134
	0.0005	46 ± 2.1	31.775	– 0.134
	0.001	47 ± 2.1	27.129	– 0.166
IIIc	0.00001	48 ± 2.1	– 45.616	– 0.114
	0.00005	48 ± 2.1	– 45.323	– 0.116
	0.0001	47 ± 2.1	– 44.701	– 0.119
	0.0005	47 ± 2.1	– 44.305	– 0.123
	0.001	46 ± 2.1	– 43.106	– 0.129

### Adsorption isotherm

The initially step of the inhibitor’s inhibitory effect on CS corrosion in 1.0 M HCl solution is mostly caused by the CGIS molecule’s adsorption through their active centers in the inhibitor’s chemical structure on the CS surface. Consequently, corrosion reactions that occur in regions devoid of CS are reduced.

**Fig. 13:**
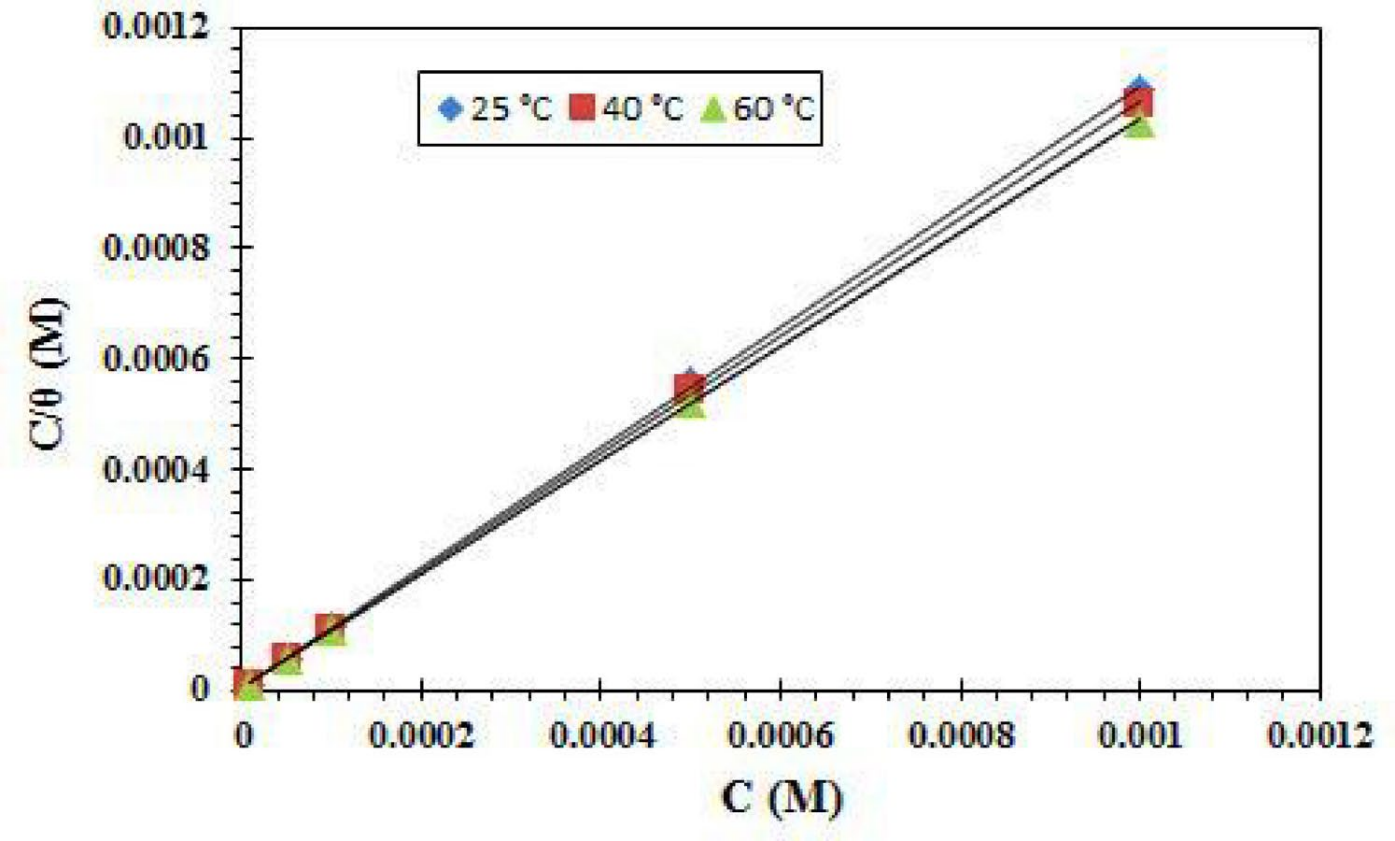
Model for Langmuir adsorption isotherm at different temperatures on CS in presence of IIIb.

To assign a convenient isotherm, we enter the values of θ into a variety of isotherms and choose the suitable one. When confirming the adsorption properties, the values of θ are quite helpful. The experimental data were fitted into various adsorption isotherm: Temkin, Langmuir and Frumkin. The Langmuir isotherm is sufficient for the CGIS molecule’s adsorption in this investigation on the CS surface as indicated by the following equation^57^:

15 $$\frac{C}{\theta}-\frac{1}{{K}_{ads}}=C$$

where K_ads_ is the adsorption equilibrium constant and C is the CGIS concentration. The C/θ vs. C relationship is shown in Fig. 13. The Langmuir isotherm’s suitability for the adsorption process was confirmed by the acquisition of a straight line with one unit slope.

The values of K_ads_ were computed using the intercept of the straight lines in the Langmuir diagram; the findings are displayed in Table 6. The CGIS molecule is significantly adsorbed onto the surface of CS, as seen by the higher value of K_ads_ in Table 6. The free energy of adsorption (∆G^o^_ads_) was computed using the following formula, which was based on the values of K_ads_ at different temperatures:

16 $$\varDelta{G}_{Ads}^{o}=-R\times T\times\mathrm{ln}\left(55.5\times{K}_{Ads}\right)$$

where 55.5% of the solution is water. Table 6 displays the thermodynamic parameters for the adsorption of the CGIS molecule on the CS surface at different temperatures in a 1.0 M HCl solution. The negative indicators of ∆G^o^_ads_ emphasize the stable and spontaneous adsorption of the CGIS molecule on the CS surface. The more beneficial ∆G^o^_ads_ promote more efficient surface coverage as the temperature rises, increasing the inhibition efficiency. The Van’t Hoff equation could be used to calculate the adsorption enthalpy:^58,59^

17 $$\mathrm{ln}\left({K}_{ads}\right)=-\frac{\varDelta{H}_{Ads}^{o}}{R\times T}+\mathrm{c}\mathrm{o}\mathrm{n}\mathrm{s}\mathrm{t}\mathrm{a}\mathrm{n}\mathrm{t}$$.

where the enthalpy of adsorption is indicated by ∆H^o^_ads_. Table 6 displays the outcomes of the ∆H^o^_ads_ values calculation. The endothermic nature of CGIS adsorption onto the CS surface is indicated by the positive ∆H^o^_ads_ value. The following formula can be used to calculate the entropy of adsorption (∆S^o^_ads_), which can then be entered into Table 6:

18 $$\varDelta{G}_{Ads}^{o}=\varDelta{H}_{Ads}^{o}-T\times\varDelta{S}_{Ads}^{o}$$

The ∆S^o^_ads_ values have positive signs which is associated with the replacement process that may be explained by the solvent’s higher entropy and the water’s higher positive entropy of desorption^60^. Another factor is a higher disruption caused by a greater amount of water molecules. One CGIS molecule might desorb it from the CS-surface^61–63^.

**Table 6:** Thermodynamic adsorption parameters.

Inhibitor name	Temp. ^o^C	K_ads_ ×10^4^ (M^− 1^)	*R* ^2^	∆G^o^_ads_ (KJ mol^− 1^)	∆H^o^_ads_ (KJ mol^− 1^)	∆S^o^_ads_ (J mol^− 1^ K^− 1^)
IIIa	25	27.778	0.9999	– 41.006	3.540	149.484
	40	29.412	0.9999	– 43.219		149.390
	60	32.258	0.9999	– 46.236		149.479
IIIb	25	20.000	0.9998	– 40.192	0.252	135.718
	40	16.667	0.9998	– 41.741		134.162
	60	20.000	0.9999	– 44.913		135.629
IIIc	25	26.316	0.9999	– 40.872	6.542	159.107
	40	26.316	0.9999	– 42.930		158.055
	60	34.483	1	– 46.421		159.047

#### The mechanism for corrosion protection

The investigated CGIS inhibitors can act as corrosion inhibitors through sticking to the surface of CS and forming a barrier through a variety of interaction mechanisms. These adsorption mechanisms include (a) The chemisorption through coordinating the lone electron pairs of the CGIS inhibitors with vacant d-orbitals of Fe, (b) The interactions of π-electrons in benzene rings of CGIS inhibitors with Fe metal surface. In addition to the quaternary ammonium (N+) group present in CGIS inhibitors form electrostatic interactions with the adsorbed chloride on the CS surface through the physical adsorption.

**Fig. 14:**
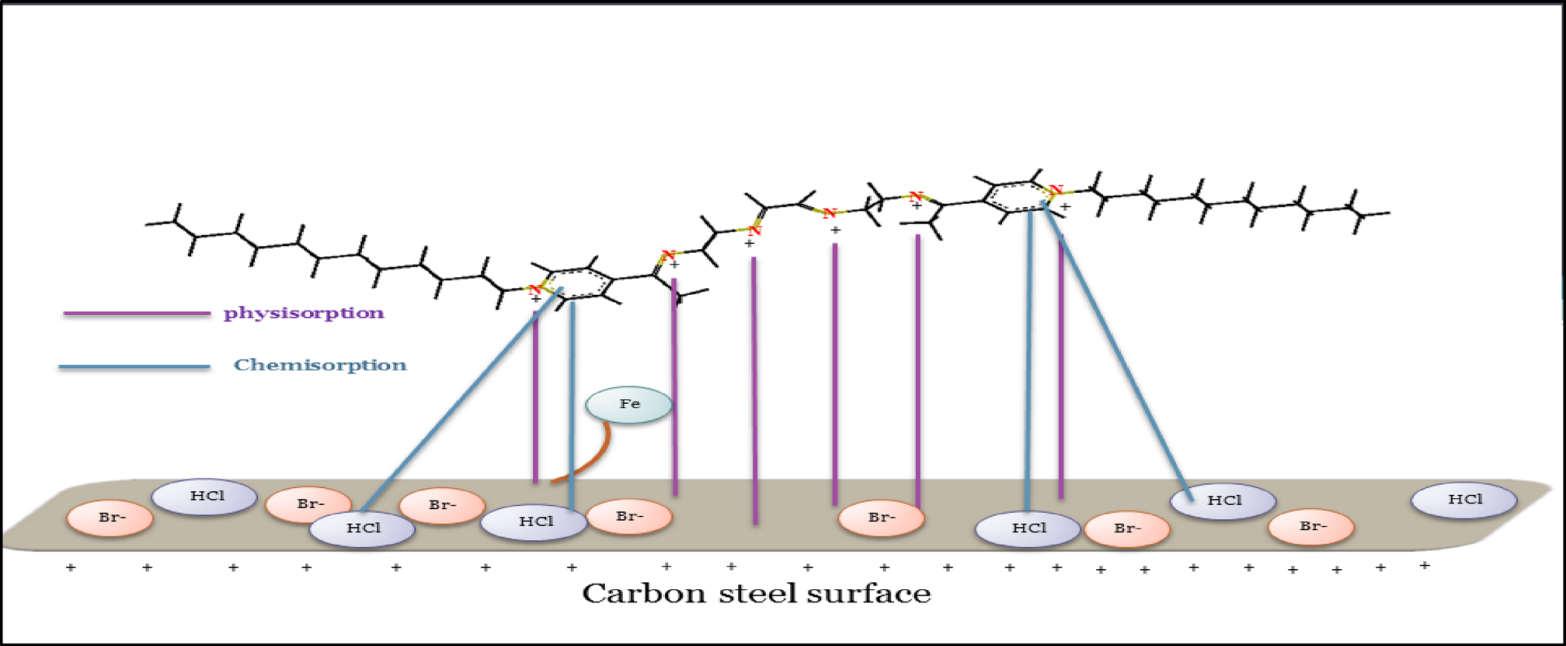
The proposed inhibition mechanism proposed by the synthesized surfactant.

Additionally, by creating an additional barrier between the CS surface and the HCl solution, the hydrophobic tails of these surfactants enhance corrosion prevention. Figure 14 illustrates the suggested mechanism for CGIS’s inhibitory activity.

### Surface characterizations via SEM/EDX

**Fig. 15:**
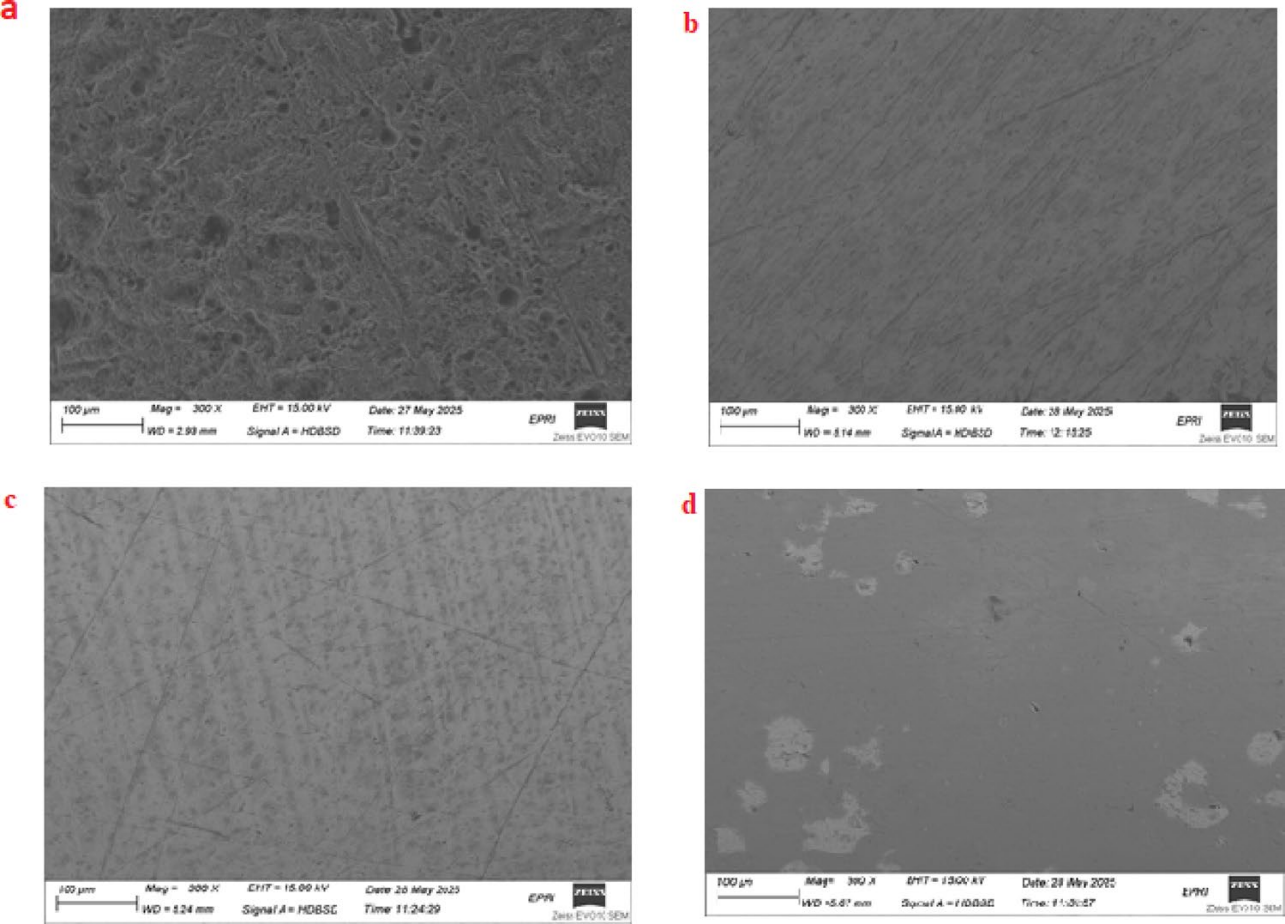
SEM photographs of steel in aggressive solution 1.0 M HCl (**a**) and steel in 1.0 M HCl containing 1 mM of IIIa (**b**), IIIb (**c**), and IIIc (**d**).

To verify the inhibitors’ adsorption on the coupon surface, the surface of the carbon steel was examined using a scanning electron microscope. The surface SEM of carbon steel with and without 1 mM of the separately generated IIIa, IIIb, and IIIc is displayed in Fig. 15. The steel’s shape following a 24-h immersion in the corrosive liquid is depicted in Fig. 15.

After being submerged in 1.0 M HCL without inhibitors, corrosion attack damaged the steel’s morphology Fig. 14. The steel surface was exceptionally smooth when the synthetic inhibitors IIIa, IIIb, and IIIc were present, suggesting that the inhibitors’ adsorption on the coupon surface had protected the carbon steel surface.

**Fig. 16:**
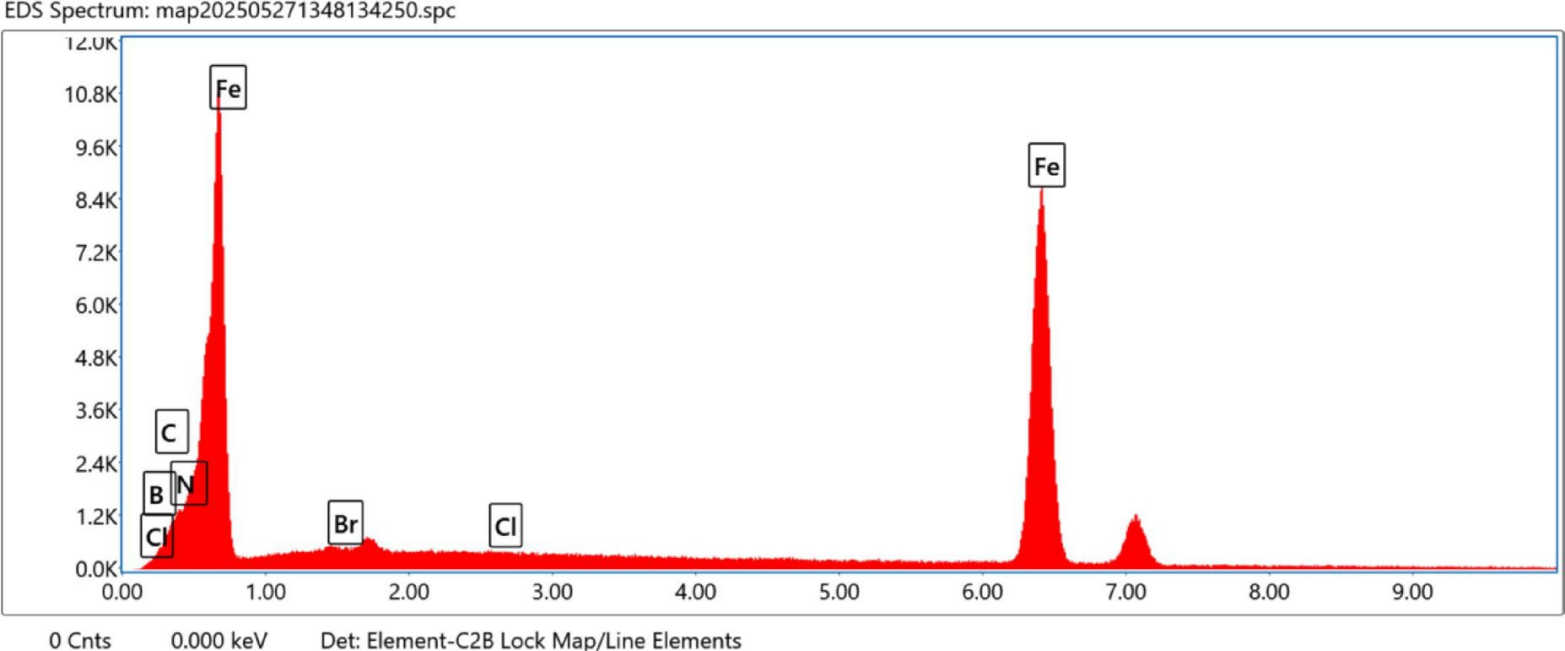
The EDX analysis for the carbon steel and quantification of elements present.

EDX gives the chemical composition the outer layer surface of carbon steel in the absence and presence of IIIa, IIIb, and IIIc with in1M HCl. The peak in tensity of Fe higher in the absence of an inhibitor while a decrease in the presence of inhibitor confirms the Shielding carbon steel surface with protecting inhibitor and decrease with corrosion product. Figure 16 show the EDX for carbon steel and quantification of elements present. The appearance of peaks of N atoms in the presence of inhibitor indicates the formation of the protective adsorbed layer.

## Conclusions

The structures of the newly synthesized long-chain cationic gemini inhibitor surfactants (IIIa, IIIb, and IIIc) were verified using FTIR and ^1^H NMR spectroscopy. The surface parameters of the prepared CGIS showed that They positioned themselves at the air/aqueous contact after being effectively absorbed. The compound IIIb with twelve carbon chain length is the most efficient surfactant and has the highest surface activity. The CGIS are effective corrosion inhibitors for CS in 1 M HCl solution where their anticorrosion efficacy rises with their concentration increase in the temperature range of 298–353 K. The adsorption of the CGIS inhibitors onto the CS-surface is chemical adsorption and follows the Langmuir isotherm. The potentiodynamic polarization (PDP) curves indicate that the synthesized CGIS is a mixed type corrosion inhibitor. The prepared CGIS inhibitors formed a protective layer onto CS-surface which is depicted by the smooth surface in SEM indicating their efficiency in mitigating corrosion. CGIS-molecules contain highly reactive centers such as azomethine group, benzene ring in addition to the quate.

## Supplementary Information

Below is the link to the electronic supplementary material.

Supplementary Material 1

## Data Availability

The datasets used and/or analyzed during the current study are available from the corresponding author on reasonable request.
