# Supplementary material for: Inhibitory influence of three new synthesized cationic gemini surfactants on the corrosion rate of carbon steel in 1 M HCl
Source: Sci Rep. 2026 Apr 9;16:12055. doi: 10.1038/s41598-026-44281-2 (PMC13068958; doi:10.1038/s41598-026-44281-2)
Supplement: Supplementary file 1 — Supplementary Material 1 [file 41598_2026_44281_MOESM1_ESM.pdf]

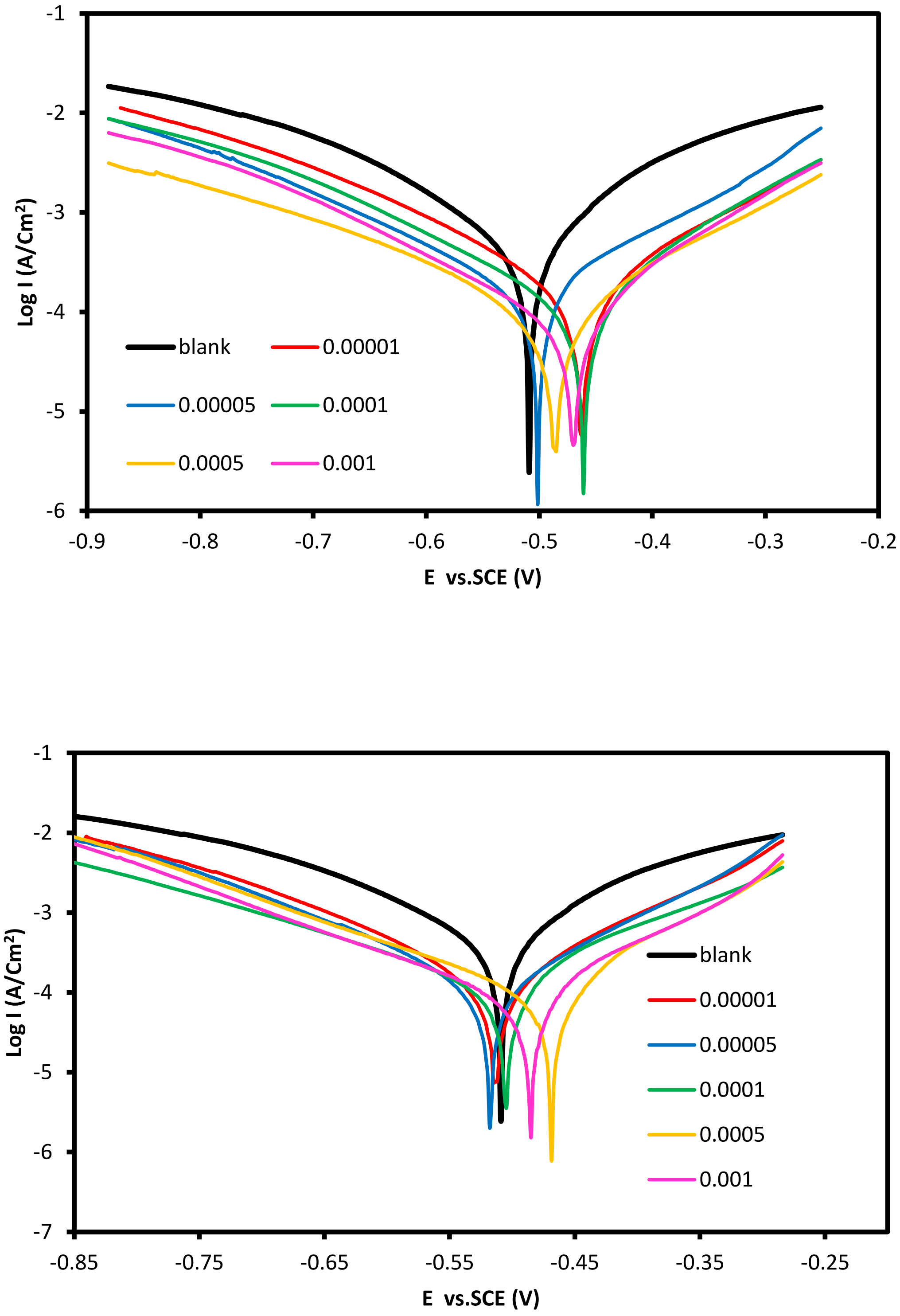

Fig. S1. Anodic and cathodic polarization curves for CS in 1.0 M HCl with and without different concentrations of IIIa and IIIc of CGIS.

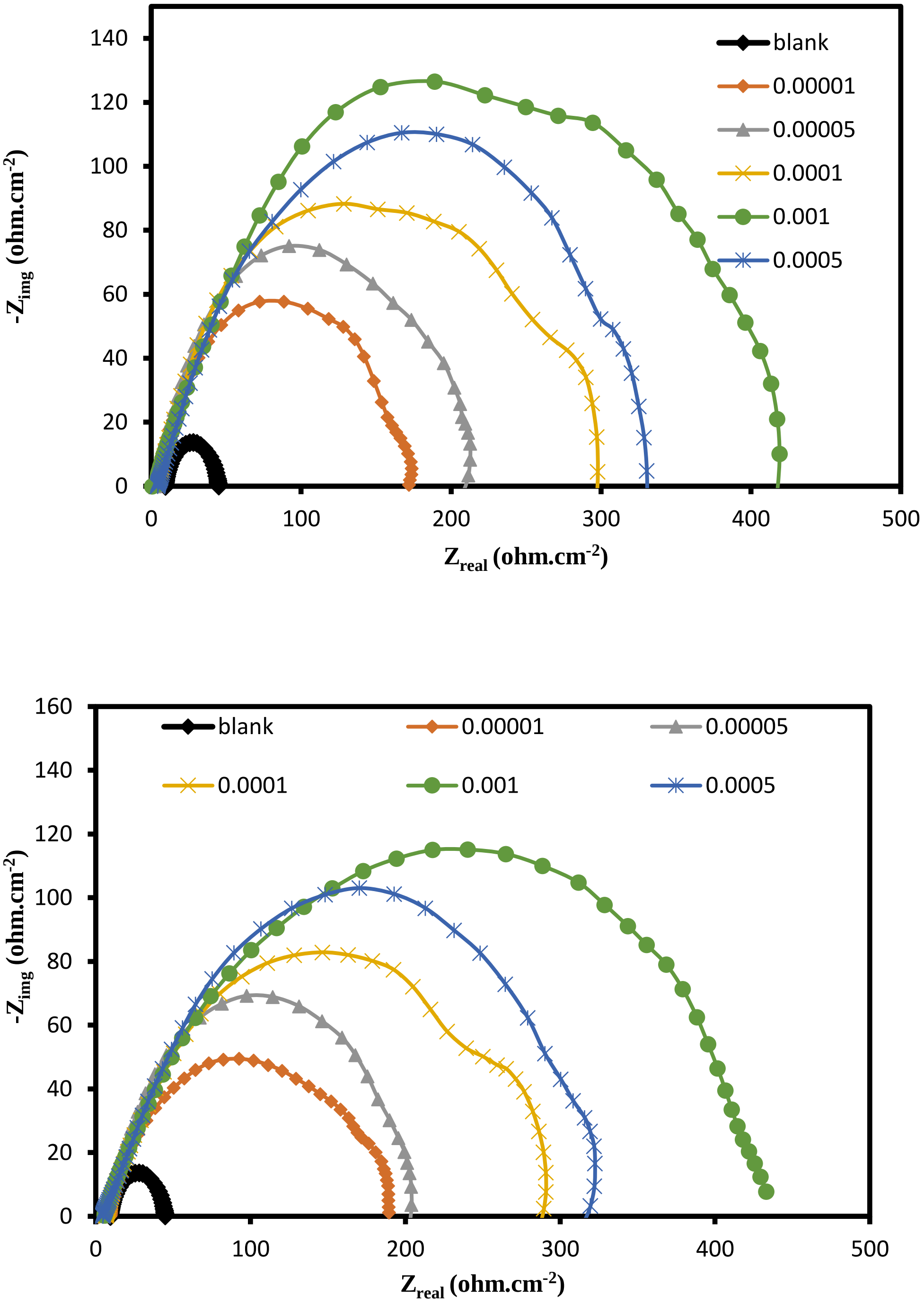

Fig. S2. Nyquist plots for CS at 25 C° in 1.0 M HCl with and without different concentrations of III_a and III_c of CGIS

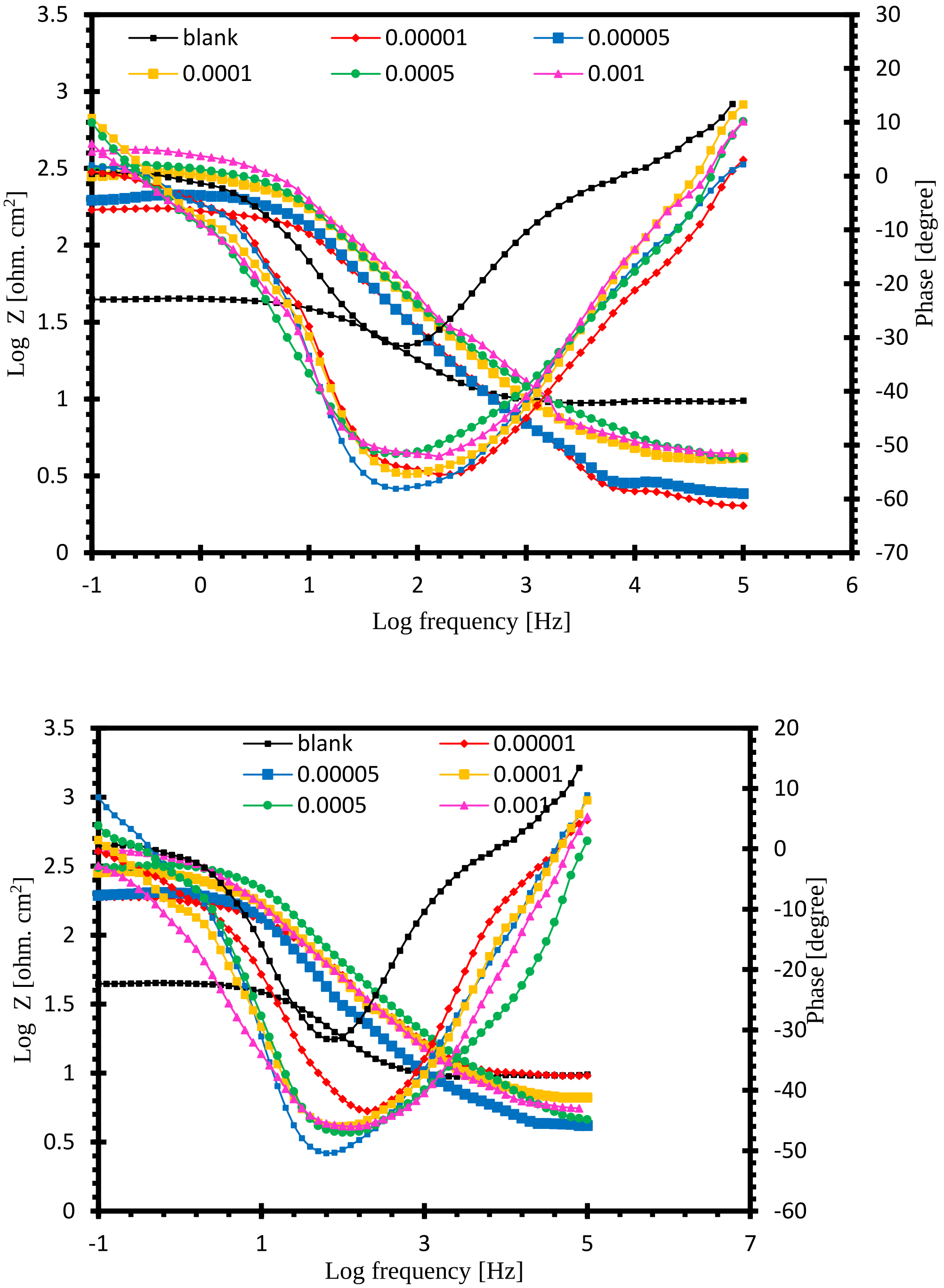

Fig. S3. Bode and phase plots for CS at 25 °C in 1.0 M HCl with and without different Concentrations of III_a and III_c of CGIS
